# Genetic Profile of Rotavirus Type A in Children under 5 Years Old in Africa: A Systematic Review of Prevalence

**DOI:** 10.3390/v16020243

**Published:** 2024-02-03

**Authors:** Sandra Miranda, Fernanda S. Tonin, Carlos Pinto-Sousa, Elsa Fortes-Gabriel, Miguel Brito

**Affiliations:** 1 Faculdade de Medicine, Universidade Agostinho Neto, Luanda, Angola; darilinga@hotmail.com (S.M.); pintodesousa@yahoo.com.br (C.P.-S.); 2 CISA-Centro de Investigação em Saúde de Angola, Caxito, Bengo, Angola; fortes.elsa@gmail.com; 3 Clínica Girassol, Luanda, Angola; 4 ESTeSL-Escola Superior de Tecnologia da Saúde, Instituto Politécnico de Lisboa, 1990-096 Lisboa, Portugal; fernanda.tonin@estesl.ipl.pt; 5 Pharmaceutical Sciences Postgraduate Program, Federal University of Paraná, Curitiba 80210-170, Brazil; 6 UPRA-Universidade Privada de Angola, Luanda, Angola; 7 ISTM- Instituto Superior Técnico Militar, Luanda, Angola

**Keywords:** rotavirus, genetic profile, children, systematic review, Africa

## Abstract

Human type A rotavirus (RV-A) is world-recognized as the major pathogen causing viral gastroenteritis in children under 5 years of age. The literature indicates a substantial increase in the diversity of rotavirus strains across continents, especially in Africa, which can pose significant challenges including an increase of disease burden and a reduction of vaccines’ effectiveness. However, few studies have mapped the variety of circulating virus strains in different regions, which may hamper decisions on epidemiological surveillance and preventive public health measures. Thus, our aim was to compile the most updated available evidence on the genetic profile of RV-A among children in Africa and determine the prevalence of different genotypes according to the geographical regions by means of a broad systematic review. Systematic searches were performed in PubMed, Scopus, Web of Science, and Scielo without language, time limits, or geographical restrictions within the African continent. We selected full-text peer-reviewed articles assessing the genetic profile (i.e., genotyping) of RV-A in children up to 5 years old in Africa. Overall, 682 records were retrieved, resulting in 75 studies included for evidence synthesis. These studies were published between 1999 and 2022, were conducted in 28 countries from the five African regions, and 48% of the studies were carried out for 24 months or more. Most studies (n = 55; 73.3%) evaluated RV-A cases before the introduction of the vaccines, while around 20% of studies (n = 13) presented data after the vaccine approval in each country. Only seven (9.3%) studies compared evidence from both periods (pre- and post-vaccine introduction). Genotyping methods to assess RV-A varied between RT-PCR, nested or multiplex RT-PCR, testing only the most common P and G-types. We observed G1 and P[8] to be the most prevalent strains in Africa, with values around 31% and 43%, respectively. Yet if all the genotypes with the following highest prevalence were added ((G1 + G2, G3, G9) and (P[8] + P[6], P[4])), these figures would represent 80% and 99% of the total prevalence. The combination G1P[8] was the most reported in the studies (around 22%). This review study demonstrated an increased strain diversity in the past two decades, which could represent a challenge to the efficacy of the current vaccine.

## 1. Introduction

Severe dehydrating diarrhea caused by rotavirus remains one of the major causes of morbidity and mortality among children under 5 years old worldwide, despite some decreasing trends in these figures in the last decade [1]. In 2019, rotavirus infections were responsible for an estimated two million hospitalizations and over 25 million outpatient visits globally. In this same year, from the over five million accounted deaths (95% CI 4.92–5.68) in children younger than 5 years, diarrhea diseases were attributed to 9.1% of cases (95% CI 7.9–9.9) and occurred mostly in low-income countries [2,3].

The genus *Rotavirus* A (RV-A) is an RNA virus (*Reoviridae* family) containing several structural viral proteins, of which VP4 (protease-cleaved protein or P protein) and VP7 (glycoprotein or G protein) strands are determinants of genetic variability and viral serotype classification (P- and G-serotypes). These proteins have been extensively studied in the past decades as targets for neutralizing antibodies, and grounded the development of live attenuated rotavirus vaccines. Between 2008 and 2009, the World Health Organization (WHO) prequalified a pentavalent bovine-human reassortant vaccine (RotaTeq27-RV5) and a monovalent vaccine based on a human RV-A strain (Rotarix-RV1). In 2018, two additional vaccines (Rotavac and ROTASIIL) were licensed, being increasingly recommended by national immunization programs [4], especially for high-risk mortality populations [5]. As of January 2022, 114 countries (including 79% of those from Africa) have introduced RV vaccination services [4,6]. In fact, a systematic review on the impact of immunization programs in sub-Saharan Africa demonstrated that the inclusion of RV1 and RV5 vaccines led to significant reductions in the proportion of positive cases in these regions from 42% (95% CI 38–46) (pre-vaccination period) to 21% (95% CI 17–25) [7].

However, it has been suggested that massive vaccination could lead to the replacement of circulating genotypes or the emergence of new variants or neutralizing antibodies escape mutants, which may reduce the effectiveness of the vaccine [8]. Moreover, a very heterogeneous distribution of genotypes of RV-A in Africa exist—and often differ from circulating strains and G-P combinations from other regions in the globe [9,10,11,12]. Additionally, dissimilar socioeconomic conditions and cultures may lead to differences in the pattern of circulation of RV-A genotypes [7]. Previous reviews conducted between 1975 and 1992 reported three quarters of rotavirus strains in Africa belonging to one of the four globally common G types circulating at that time, namely serotypes G1, G2, G3, or G4 [13]. Later studies showed that the genotypes G1, G2, G3, G9, and G12 were the most common, together with P[8], P[6], and P[11]. It seems that the combinations G1P[8], G2P[4], G3P[8], G4P[8], and G9P[8] are responsible for around 90% of all RV-A infections in the continent [9,14,15].

Yet, the literature lacks further synthesized and more updated evidence on the genetic profile of RV-A in African countries, especially for the pediatric population. Only one systematic review, without a published protocol (2017) and assessing the genotype profile of the virus in Africa during 2006–2016, has been published [13,16]. Thus, we aimed to compile the current evidence on the genetic profile of RV-A in children up to 5 years old living in Africa and determine the prevalence of the genotypes according to the different regions by means of a broad systematic review.

## 2. Materials and Methods

A systematic review to synthesize the pooled prevalence of circulating RV-A in children under 5 in Africa was performed and reported according to the Preferred Reporting Items for Systematic Reviews and Meta-Analyses—PRISMA guidelines and Cochrane Collaboration recommendations [17,18]. The protocol of this systematic review was registered in the international prospective register of systematic reviews—PROSPERO (CRD42022346530)—and is available at Open Science Framework (DOI 10.17605/OSF.IO/RSZC6). Two authors conduct, independently, all steps of this studies’ selection and data extraction. Disagreements were resolved by discussion with a third author arbitrating in the circumstance of unresolved discrepancies.

### 2.1. Search Strategy

This study review was conducted by searching the following electronic databases for primary peer-reviewed studies: MEDLINE (PubMed), Scopus, Web of Science, and Scielo (updated searches in December 2022). The search was not limited by any filter tool, language, or country. Trial registration databases (www.clinicaltrials.gov; accessed on 10 December 2022), internet-based relevant databases (WHO Global Health Library, which encompasses African Index Medicus), and the reference lists of the included studies were also searched as part of the manual searching process. A comprehensive search strategy was developed using subject headings related to four sets of descriptors (rotavirus, genotyping, children, Africa), combined with Boolean Operators AND and OR. The search strategies adapted for each database are available in the Supplemental Materials (Table S1). 

### 2.2. Study Selection

Records retrieved from the databases were exported to a reference management program (EndNote version X9.2, Clarivate, London, UK) where duplicates were removed. Thereafter, the management of references and data extraction used Excel sheets (Microsoft 2020, Redmond, WA, USA). Titles and abstracts of the studies were independently screened by two authors to remove irrelevant records. The full text of potentially eligible studies was retrieved and independently assessed for eligibility by two of the authors. The three inclusion criteria were (i) peer-reviewed primary articles reporting data on the genetic profile (genotyping) of RV-A; (ii) articles including children under 5 years old; (iii) studies carried out in at least one African country. Studies conducted in other populations (adults or children older than 5 years), in vitro or in vivo studies, as well as those without clear evidence of the type of technique used for genotyping were excluded. Discussion papers, letters, editorials, reviews, and articles in non-Roman characters were also excluded.

### 2.3. Data Extraction and Quality Assessment

A standardized form in Excel sheets (Microsoft 2020, Redmond, WA, USA) was used to extract information on: articles’ general data (author’s name, year of publication, country, sample size, study duration); participants and their characteristics (age, in- or outpatients); genotyping methods used, number of samples tested, absolute numbers and percentages for the relevant genotypes. Whenever necessary, indirect data from figures and charts were collected.

The methodological quality of the included studies was assessed by means of the JBI—Jonna Briggs Institute Critical Appraisal tool [16] for the domains of appropriateness of study design, selection bias, appropriate statistical analysis, and presentation of study findings. For each study, the grading of each component and the global study rating was assigned to the categories: low (<5), moderate (5–6), and high quality (7–8). To assign the final quality score, authors also verified if the stated objectives of the paper matched the reporting of outcomes within the paper (Supplementary Figure S1).

### 2.4. Statistical Analysis and Synthesis

A narrative synthesis of the findings from the included studies, structured around the population characteristics, geographical region, and genotype profiling, was developed. Prevalence calculations of identified genotypes were performed (i.e., by dividing the number of positive cases of a given genotype by the number of samples tested) and reported with a 95% confidence interval (95% CI—upper and lower limits) (Comprehensive Meta-analysis software version 3.0; Microsoft Excel). Location maps of G and P genotypes for the six African regions of the United Nations were built. Analyses were performed in IBM SPSS statistics version 26 software.

## 3. Results

**Figure 1:**
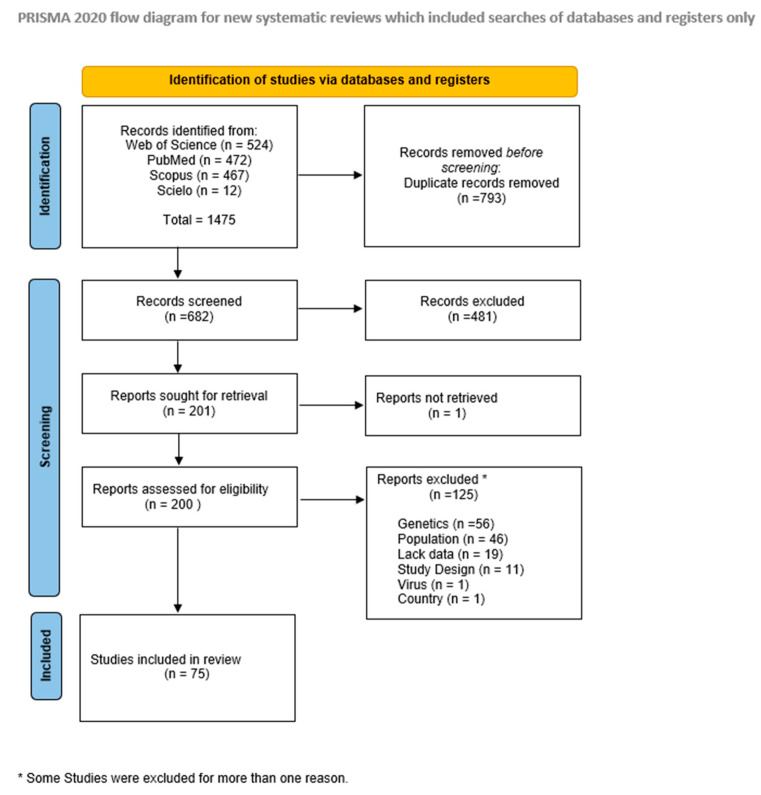
Study selection flow diagram.

Overall, 682 records were retrieved from the databases after duplicates removal, from which 200 were fully assessed during the eligibility phase, resulting in 75 studies included for evidence synthesis (see Figure 1).

**Figure 2:**
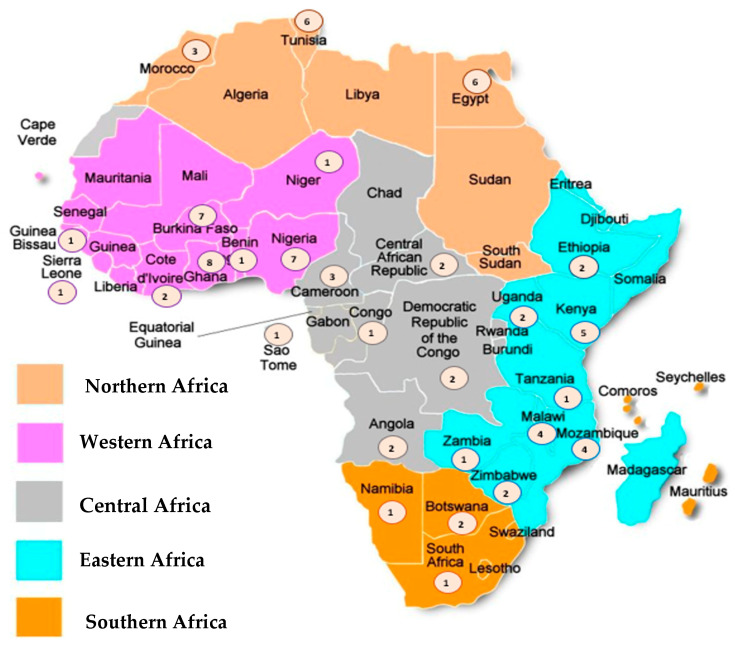
Geographical distribution of the studies. Note: The circles represent the number of studies included for each country.

These studies were published between 1999 and 2022 and were conducted in 28 countries located in the five African regions, namely: 25 (33.3%) studies assessing the Western region, 21 (28.0%) from the Eastern region, 15 (20.0%) from the Northern region, 11 (14.7%) from the Central Region, and 3 (4.0%) studies from the Southern region of the continent (Figure 2). Around half (n = 36; 48%) of the studies were performed for 24 months or more, the total sample size generally being less than 250 patients. Most studies (n = 31; 41.3%) evaluated RV-A cases in hospital settings, while outpatient department visits were reported in 30.6% of the studies (n = 23); children attending any of these settings were assessed by n = 21; 28% of studies. Most studies (n = 55; 73.3%) evaluated RV-A cases before the introduction of the vaccines in the respective countries (vaccine introduction dates in Supplementary Table S2), while around 20% of studies (n = 13) presented data after the vaccine approval in each country. Only seven (9.3%) studies compared evidence from both periods (pre-and post-vaccine introduction). Genotyping methods to assess RV-A varied between RT-PCR, nested or multiplex RT-PCR. Most studies tested only the most common P and G-types (Table 1).

A total of 17,418 genotyped samples were analyzed for prevalence of various RV-A genotypes, 14,759 strains being characterized for the G specificity, 14,258 for P specificity, and 13,003 for both P and G antigens. Considering the very low incidence of other non-typeable RV strains and mixed infections, they were not included in the final synthesis.

**Figure 3:**
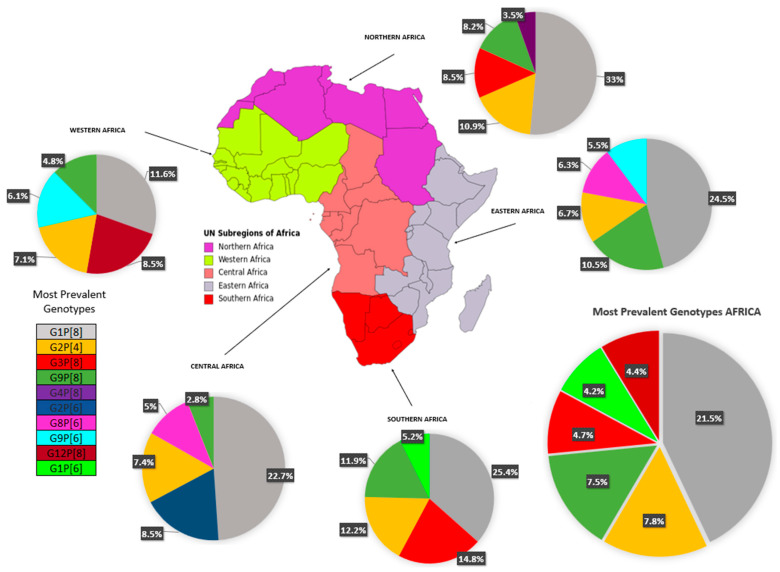
Circulating rotavirus strains in the African regions.

Table 2 depicts the complete data on RV-A genotypes from 28 of 54 African countries (51.8%) and Figure 3 summarizes the evidence distributed according to the regions of the African continent. Overall, G1 was the most prevalent strain (n = 5352 cases; 30.73% [95% CI 24.2–37.2]), with the highest prevalence in the North region (40.5% [95% CI 33.9–47.1]), followed by G2 (n = 2588 cases; 14.9% [95% CI 11.0–18.7]), found especially in the West (18.5%) and Central (18.1%) regions. The rotavirus G3 strain represented the third most prevalent strain (11.0%, [95% CI 5.6–19.1]), being most detected in the South (20%) (see Table 2). Regarding P genotypes, P[8] strains were highly reported (n = 7465 cases; 42.8% [95% CI 33.0–52.7]) in all regions, followed by P[6] (24.9%) and P[4] (13.7%) strains. The most prevalent combination was G1P[8] (n = 3745 cases; 21.5% [95% CI 12.9–30]), with rates ranging from 11.6% in the West region to 33.0% in the North. Other combined strains such as G2P[4] (n = 1364; 7.8% [95% CI 5—10.6]) and G9P[8] (n = 1309; 7.5% [95% CI 3.3–11.7]) were also fairly reported.

After vaccine introduction, G1P[8] continued to be the most prevalent (20.4%) globally, due to the Northern (51.7%) and Eastern (24.3%) regions. In the Western region, G12P[8] was the most prevalent (23%), and G2P[4] was most prevalent in the Southern region (27.3%). A small number of post-vaccine studies (13), and the absence of studies from the central region may have created an important bias in the regional analysis.

The methodological quality assessment of studies demonstrated most reports as high and medium quality. Of the assessed domains, the identification of limitations, confounding factors, strategies to deal with limitations, and the lack of next steps were the most problematic. These aspects should be improved in future reports (full assessment in Supplemental Materials).

## 4. Discussion

This systematic review synthesized and critically assessed the evidence of 75 primary studies on RV-A genotypes in children under 5 in Africa over a 23-year period and demonstrated the prevalence of the infection is still commonplace. This highlights the need of further measures for reducing the health, social, and economic burdens related to this condition in the continent—including the development of vaccines and immunization programs targeting circulating genotypes.

We observed G1 and P[8] to be the most prevalent strains in Africa, with values around 31% and 43%, respectively. Yet if all the genotypes with the following highest prevalence were added, (G1 + G2, G3, G9) and (P[8] + P[6], P[4]), these figures would represent 80% and 99% of the total prevalence. The combination G1P[8] was the most reported in the studies (around 22%). Previous studies similarly reported these genotypes as commonly found in the continent (comparable prevalence rates varying from 25 to 45% for G1 and P[8]), yet their occurrence may significantly differ across geographical regions worldwide [13,14,90,91]. For instance, higher rates of G1P[8] infection have been found in North America, Europe, and Australia (70% of all circulating strains), while this figure is about 30% in South America and Asia [91].

We additionally identified a potential increasing trend in the proportion of novel strains, such as G12 (7.2%) and G8 (5.9%), but also the combinations G2P[4] (7.8%) and G9P[8] (7.5%), that have been similarly detected globally [21]. Moreover, we found a fair prevalence (3–5%) of unusual strain combinations such as G1P[6], G3P[8], G2P[6], G3P[8], G12P[8], and G8P[6] that may reflect the diversity and nature of RV-A in Africa and its unique distribution compared with other regions. The identification of uncommon genotypes such as G5 (0.01%), G6 (0.6%), and G10 (0.8%) raise further awareness about the heterogeneity of RV-A in Africa and the need for active epidemiological surveillance among children under 5.

This review study demonstrated an increased strain diversity in the past two decades, when compared with data from before 1997–2006 in Africa [11]. As studies suggested, this increase in strain diversity can be explained by the introduction and prevalent use of more sensitive RT-PCR genotyping methods that enable the detection of common and uncommon strain types, lending multiple infections and rotavirus evolution in vivo, and that common strains may have evolved by genetic drift [11].

A number of rotavirus strains remained non-typeable (14.5%); less than the 16% in a review study carried out in Africa from 1997 to 2006, and more likely than the 14.8% found globally [11], allowing the possibility that other serotypes have not yet been identified.

Even though the most common G/P combinations reported in different geographical regions appear similar, the proportions vary per geographic region and over time.

The complexity of the molecular epidemiology of rotavirus strains and its variability was shown in this review study, with only 13 post-vaccine introduction studies conducted in four African regions, and the predominant genotypes varying in the regional analysis with an overall predominant prevalence being G1P[8] (20.4%). The recognition that the circulating strains will fluctuate over time and in different regions of the continent is important for the monitoring of strain diversity in the period after rotavirus vaccines introduction.

In the analysis of the relevance of strain diversity to rotavirus vaccine programs, the current oral, live attenuated rotavirus vaccines have greatly reduce the burden of severe rotavirus disease in Africa. These include a monovalent human rotavirus G1P[8] vaccine (Rotarix) and a (RotaTeq) pentavalent human-bovine reassortant vaccine that covers serotypes G1, G2, G3, G4, and P[8]. It will be important to demonstrate vaccine efficacy in settings where strains share neither G or P type with these vaccines. In this review, of the single rotavirus strains examined, 12.7% did not share either G or P type with RotaTeq and 29% did not share a G or P type with Rotarix. However, many other factors may be involved in the protective immunity and further study is needed in less developed settings to study the cross-protection of non-vaccine strains [8,92,93,94,95].

This study has some limitations, as data were available from only 51.8% (28/54) of the African countries and only 13 studies were conducted after the vaccine introduction. Also, the majority of the studies only detect common G and P-types and sequencing tests were performed in very few studies, probably because of not typing strains. Although the studies included in this review provide an indication of genotypes circulating throughout the African continent, they may not represent all countries in the region. Different definitions of genotypes, as well as the consideration of a wide variety of sequencing techniques of the rotavirus, whose numbers of eligible studies may be limited, may affect the characterization of some genotypes.

## 5. Conclusions

This systematic review compiled the most recent findings from primary studies of genetic identification of RV-A circulating in Africa in the past 23 years and presented the pooled prevalence of circulating rotavirus genotypes in children under 5 years old in the five African geographic regions.

In the African continent, 43 of 54 (79.6%) countries have introduced rotavirus vaccines into their immunization programs [96], representing over half of all countries in the world [8].

The high prevalence of mixed infections observed in this study as well as other studies in the continent [57] constitutes an optimal moment for the reassortment of the rotavirus genome that can lead to the generation of new rotavirus strains and may generate new genome constellations that allow rotavirus type A to expand its host range or evade immune responses [97]. The diversity of rotavirus strains in the continent, that carry a higher burden of rotavirus mortality, could represent a challenge to the efficacy of current vaccines.

African surveillance studies post-vaccine introduction are crucial to understanding the impact of the vaccine on rotavirus circulating strains, and assure vaccine efficacy. It is fundamental to maintain an efficient rotavirus surveillance network and update the information of circulating strains in each country of the African region.

We, thus, believe that our findings may directly contribute towards the updating of evidence on rotavirus circulating strains, contributing to the development of next generation rotavirus vaccines, surveillance mechanisms, and health policy measures.

## Figures and Tables

**Table 1:** Summary of the studies’ characteristics.

Author (Data of Publication)	Country	Year (s) of Sample Collections	Genotyping Method	Nº of Genotyped Samples	Vaccine Introduction Period ^a^
Naficy A.B., et al. [19]	Egypt	1995–1996	RT-PCR	46	Before
Allayeh A.K., et al. [20]	Egypt	2015–2016	RT-PCR	37	Before
Saudy N., et al. [21]	Egypt	2010–2012	Multiplex RT-PCR	45	Before
Elnady H.G., et al. [22]	Egypt	2012–2012	RT-PCR	53	Before
Matson D.O., et al. [23]	Egypt	2000–2002	Nested RT-PCR	243	Before
Ahmed S.F., et al. [24]	Egypt	2004–2007	Nested, multiplex RT-PCR	164	Before
Benhafid M., et al. [25]	Morocco	2006–2007	RT-PCR	134	Before
Benhafid M., et al. [26]	Morocco	2006–2009	Semi-nested, multiplex RT-PCR	548	Before
El Qazoui M., et al. [27]	Morocco	2011	Multiplex RT-PCR	89	After
Chouikha A., et al. [28]	Tunisia	2005–2007	Semi-nested, multiplex RT-PCR	323	Before
Trabelsi A., et al. [29]	Tunisia	2000–2003	Semi-nested multiplex RT-PCR	63	Before
Soltani M., et al. [30]	Tunisia	2009–2011	Semi-nested, multiplex RT-PCR	188	Before
Chouikha A., et al. [31]	Tunisia	2005–2007	Semi-nested, multiplex RT-PCR	180	Before
Moussa A., et al. [32]	Tunisia	2009–2014	Semi-nested RT-PCR	270	Before
Bennour H., et al. [33]	Tunisia	2015–2017	Multiplex RT-PCR	72	Before
Agbla J.M., et al. [34]	Benin	2016–2018	Multiplex RT-PCR	186	Before
Steele A.D., et al. [35]	Burkina Faso	1994	RT-PCR	36	Before
Bonkoungou I.J., et al. [36]	Burkina Faso	2008–2010	Semi-nested multiplex RT-PCR	140	Before
Rönnelid Y., et al. [37]	Burkina Faso	2015–2015	Multiplex RT-PCR	20	After
Nordgren J., et al. [38]	Burkina Faso	2010	Semi-nested, multiplex RT-PCR	56	Before
Nordgren J., et al. [10]	Burkina Faso	2009–2010	Semi-nested, multiplex RT-PCR	100	Before
Bonkoungou I.J.O., et al. [39]	Burkina Faso	2012–2013	RT-PCR	67	Before
Armah G.E., et al. [40]	Ghana	1999	RT-PCR	46	Before
Asmah R.H., et al. [41]	Ghana	1998	Semi-nested, multiplex RT-PCR	50	Before
Binka F.N., et al. [42]	Ghana	1998–2020	Semi-nested PCR	238	Before
Enweronu-Laryea C.C., et al. [43]	Ghana	2007–2011	RT-PCR	876	Before
Lartey B.L., et al. [44]	Ghana	2009–2016	RT-PCR	1363	Before and After
Letsa V., et al. [45]	Ghana	2014–2016	Semi-nested, multiplex RT-PCR	136	After
Damanka S., et al. [46]	Ghana	2004–2005	RT-PCR	70	Before
Nielsen N.M., et al. [47]	Guinea-Bissau	2002	Multiplex RT-PCR	104	Before
Boni-Cisse C., et al. [48]	Ivory Coast	2010–2013	Multiplex RT-PCR	186	Before
Page A.L., et al. [49]	Niger	2010–2013	RT-PCR	449	Before
Audu R., et al. [50]	Nigeria	1996–1997	RT-PCR	23	Before
Ianiro G., et al. [51]	Nigeria	2013	nested RT-PCR	66	Before
Ayolabi C.I. [52]	Nigeria	2007–2008	RT-PCR	58	Before
Uzoma E.B., et al. [53]	Nigeria	2012–2013	Nested, multiplex RT-PCR	49	Before
Japhet M.O., et al. [54]	Nigeria	2012–2013	Semi-nested, multiplex RT-PCR	49	Before
Amadu D.O., et al. [55]	Nigeria	2013–2014	Multiplex RT-PCR	25	Before
Jere K.C., et al. [56]	Sierra Leone	2005	RT-PCR	43	Before
Armah G.E., et al. [57]	Burkina Faso; Ivory Coast; Ghana, Nigeria, Cameroon.	1996–2000	RT-PCR	925	Before
Esteves A., et al. [15]	Angola	2012–2013	Semi-nested, multiplex RT-PCR	116	Before
Gasparinho C, et al. [9]	Angola	2012–2013	Semi-nested, multiplex RT-PCR	72	Before
Esona M.D., et al. [58]	Cameroon	1999–2000	RT-PCR	89	Before
Boula A., et al. [59]	Cameroon	2007–2012	Semi-nested, multiplex RT-PCR	898	Before
Ndze V.N., et al. [60]	Cameroon	2010–2011	RT-PCR	135	Before
Banga-Mingo V., et al. [61]	CAR	2011–2013	Multiple RT-PCR	160	Before
Moure U.A.E., et al. [62]	CAR	2014–2016	Semi-nested, multiplex RT-PCR	100	Before
Mayindou G., et al. [63]	Congo	2012–2013	RT-PCR	219	Before
Kabue J.P., et al. [64]	DRC	2003–2005	RT-PCR	119	Before
Pukuta E.S., et al. [65]	DRC	2009–2012	Multiplex RT-PCR	330	Before
Istrate C., et al. [66]	São Tome and Principe	2011	Semi-nested, multiplex RT-PCR	83	Before
Abebe A., et al. [67]	Ethiopia	2007–2012	Semi-nested, multiplex RT-PCR	215	Before
Gelaw A., et al. [68]	Ethiopia	2015–2016	RT-PCR	125	After
Nyangao J., et al. [69]	Kenya	2000–2002	Nested RT-PCR	108	Before
Wandera E.A., et al. [70]	Kenya	2009–2014	Semi-nested, multiplex RT-PCR	429	Before
Raini S.K., et al. [71]	Kenya	2012–2013	Nested RT-PCR	30	Before
Kiulia N.M., et al. [72]	Kenya	2009–2011	RT-PCR	157	Before
Wandera E.A., et al. [73]	Kenya	2011–2016	Semi-nested, multiplex RT-PCR	61	Before and After
Cunliffe N.A., et al. [74]	Malawi	1997–1998	Semi-nested, multiplex RT-PCR	100	Before
Cunliffe N.A., et al. [75]	Malawi	1997–1999	Multiplex RT-PCR	414	Before
Cunliffe N.A., et al. [76]	Malawi	1997–1999	RT-PCR	1130	Before
Turner A., et al. [77]	Malawi	2008–2009	Semi-nested, multiplex RT-PCR	220	Before
João E.D., et al. [78]	Mozambique	2015–2019	RT-PCR	650	Before and After
João E.D., et al. [12]	Mozambique	2012–2013	Semi-nested, multiplex RT-PCR	157	Before
Chissaque A., et al. [79]	Mozambique	2015–2019	RT-PCR	152	Before and After
Manjate F., et al. [80]	Mozambique	2008–2012 2016–2019	Semi-nested, multiplex RT-PCR	291	Before and After
Hokororo A., et al. [81]	Tanzania	2010–2012	Multiplex RT-PCR	100	Before
Odiit A., et al. [82]	Uganda	2006–2012	Semi-nested, RT-PCR	354	Before
Bwogi J., et al. [83]	Uganda	2012–2013	Nested RT-PCR	204	Before
Simwaka J., et al. [84]	Zambia	2016	RT-PCR	116	After
Mukaratirwa A., at al. [85]	Zimbabwe	2008–2016	RT-PCR	1096	Before and After
Mukaratirwa A., et al. [86]	Zimbabwe	2008–2011	RT-PCR	127	Before
Mokomane M., et al. [87]	Botswana	2011–2018	Multiplex RT-PCR	284	Before and After
Page N., et al. [88]	Namibia	1998–1999	RT-PCR	113	Before
Seheri L.M., et al. [89]	South Africa	2003–2006	Semi-nested, RT-PCR	648	Before

Notes. ^a^ Vaccine introduction dates for each country are in Supplementary Table S2. The full information can be found in the Supplementary Table S3.

**Table 2:** Rotavirus A genotypes in Africa.

Genotype	Positive Cases	African Regions—Prevalence (%)					
		North	West	Central	East	South	Global [95% CI]
G1	5352	40.5	24.8	34.3	29.8	34.9	30.7 [24.2–37.3]
G2	2588	16.2	18.5	18.1	10	14.7	14.9 [11.0–18.7]
G3	1899	11.5	12.9	8.4	8.4	20.0	10.9 [5.6–16.2]
G4	282	4.9	0.9	1.8	1.2	-	1.6 [0–4.2]
G5	2	-	0.02	0.04	-	-	0.01 [0–0.1]
G6	107	-	1.2	1.7	0.05	-	0.6 [0–2.4]
G8	1042	0.2	2.4	6.9	11.4	3.3	6.0 [1.1–10.9]
G9	2086	9.1	8.3	3.7	19.0	14.5	12.0 [5.4–18.6]
G10	140	-	2.5	0.3	-	-	0.80 [0–10.6]
G12	1261	0.8	10.7	9.3	6.7	3.3	7.2 [2.7–11.8]
							
P (4)	2390	338	672	213	1012	155	13.72 [10.62–16.82]
P (6)	4353	190	1559	902	1520	182	24.99 [12.35–37.64]
P (8)	7465	1329	1856	936	2770	574	42.86 [33.01–52,71]
P (9)	12	2	7	3	-	-	0.07 [0.01–0.12]
P (10)	7	-	6	1	-	-	0.04 [−0.28–0.36]
P (11)	30	29	-	1	-	-	0.17 [−4.95–5.29]
P (14)	1	1	-	-	-	-	0.01 [0.01]
							
G1P[4]	114	31	15	28	37	3	0.65 [0.1–1.2]
G1P[6]	742	53	218	202	215	54	4.26 [1.6–6.8]
G1P[8]	3745	811	614	526	1529	265	21.50 [12.9–30]
G2P[4]	1364	267	376	177	416	128	7.83 [5–10.6]
G2P[6]	709	40	306	205	132	26	4.07 [0.8–7.4]
G2P[8]	143	16	74	34	19	-	0.82 [0.06–1.6]
G3P[4]	292	26	112	2	142	10	1.68 [0.7–2.7]
G3P[6]	516	10	324	39	99	44	2.96 [0.3–5.5]
G3P[8]	815	209	62	52	337	155	4.68 [1.4–10.8]
G4P[8]	163	85	10	12	56	-	0.94 [−1.12- 3]
G8P[4]	238	-	7	9	211	11	1.37 [−0.68–3.41]
G8P[6]	551	-	32	120	390	9	3.16 [−0.79–7.12]
G8P[8]	104	-	14	19	56	15	0.60 [−0.06–1.26]
G9P[4]	125	1	36	2	83	3	0.72 [0.12–1.31]
G9P[6]	473	20	70	15	343	25	2.72 [0.49–4.94]
G9P[8]	1309	202	259	67	657	124	7.52 [3.3–11.73]
G10P[6]	110		110	-	-	-	0.63 [0.63]
G12P[6]	389	13	68	78	210	20	2.23 [0.87–3.6]
G12P[8]	772	10	457	131	160	14	4.43 [0.69–8.17]
Others *	329	52	112	74	91	-	1.89 [0.62–3.15]

Notes. Number of genotyped samples: 17,418. Prevalences are presented in percentages. * Others Genotypes-regional numbers (Global Prevalence < 0.5%): G1P[9] West 3; G1P[10] Central 1; G1P[11] North 5; G2P[11] North 7; G3P[9] Central 1; G3P[11] North 10; G4P[4] North 2, West 18, Central 2, East 4; G4P[6] North 23, West 10, Central 27, East 12; G4P[11] North 1; G5P[8] West 1, Central 1; G6P[6] West 58, Central 26; G6P[8] North 2, West 1, Central 3; G8P[14] North 1; G9P[10] West 2; G9P[11] North 1; G10P[4] North 1; G10P[8] West 13, Central 8; G12P[4] West 4, Central 5, East 75.

## Data Availability

The authors confirm that the data supporting the findings of this study are available within the article [and/or] its Supplemental Materials.

## Supplementary Materials

The following are available online at https://www.mdpi.com/article/10.3390/v16020243/s1, Table S1: Search Strategies; Table S2: Characteristics of the Included Studies; Table S3: Vaccine Introduction Dates-African Countries (October 2023); Figure S1: Critical Appraisal of the Included Studies.

## References

[1] Bernadeta D., Hannah R., Max R. (2019). Diarrheal Diseases—Our World in Data November 2019. https://ourworldindata.org/diarrheal-diseases#burden-of-diarrheal-diseases.

[2] Perin J., Mulick A., Yeung D., Villavicencio F., Lopez G., Strong K.L., David P.-M., Cousens S., Black R.E., Liu L. (2022). Global, regional, and national causes of under-5 mortality in 2000–19: An updated systematic analysis with implications for the Sustainable Development Goals. Lancet Child Adolesc. Health.

[3] Damtie D., Melku M., Tessema B., Vlasova A.N. (2020). Prevalence and Genetic Diversity of Rotaviruses among under-Five Children in Ethiopia: A Systematic Review and Meta-Analysis. Viruses.

[4] Manual for the Surveillance of Vaccine-Preventable Diseases|CDC. https://www.cdc.gov/vaccines/pubs/surv-manual/index.html.

[5] World Health Organization (2021). Rotavirus vaccines: WHO position paper—July 2021—Vaccines antirotavirus: Note de synthèse de l’OMS—Juillet 2021. Wkly Epidemiol. Rec..

[6] GBD 2019 Diseases and Injuries Collaborators (2020). Global burden of 369 diseases and injuries in 204 countries and territories, 1990–2019: A systematic analysis for the Global Burden of Disease Study 2019. Lancet.

[7] Godfrey O., Zhang W., Amponsem-Boateng C., Oppong T.B., Zhao Q.L., Li D. (2020). Evidence of rotavirus vaccine impact in sub-Saharan Africa: Systematic review and meta-analysis. PLoS ONE.

[8] Technical Resources|Rota Council. https://preventrotavirus.org/resources/technical-resources/.

[9] Gasparinho C., Piedade J., Mirante M.C., Mendes C., Mayer C., Nery S.V., Brito M., Istrate C. (2017). Characterization of rotavirus infection in children with acute gastroenteritis in Bengo province, Northwestern Angola, prior to vaccine introduction. PLoS ONE.

[10] Nordgren J., Nitiema L., Sharma S., Ouermi D., Traore A.S., Simpore J., Svensson L. (2012). Emergence of Unusual G6P[6] Rotaviruses in Children, Burkina Faso, 2009–2010. Emerg. Infect. Dis..

[11] Todd S., Page N.A., Steele A.D., Peenze I., Cunliffe N.A. (2010). Rotavirus Strain Types Circulating in Africa: Review of Studies Published during 1997–2006. J. Infect. Dis..

[12] João E.D., Strydom A., O’neill H.G., Cuamba A., Cassocera M., Acácio S., Mandomando I., Motanyane L., Page N., de Deus N. (2018). Rotavirus A strains obtained from children with acute gastroenteritis in Mozambique, 2012-2013: G and P genotypes and phylogenetic analysis of VP7 and partial VP4 genes. Arch. Virol..

[13] Cunliffe N.A., Kilgore P.E., Bresee J.S., Steele A.D., Luo N., Hart C.A., Glass I.R. (1998). Epidemiology of rotavirus diarrhoea in Africa: A review to assess the need for rotavirus immunization. Bull. World Health Organ..

[14] Ouermi D., Soubeiga D., Nadembega W., Sawadogo P., Zohoncon T., Obiri-Yebo D., Djigma F., Nordgren J., Simpore J. (2017). Molecular Epidemiology of Rotavirus in Children under Five in Africa (2006–2016): A Systematic Review. Pak. J. Biol. Sci..

[15] Esteves A., Nordgren J., Pereira J., Fortes F., Dimbu R., Saraiva N., Mendes C., Istrate C. (2016). Molecular epidemiology of rotavirus in four provinces of Angola before vaccine introduction. J. Med. Virol..

[16] Aromataris E., Munn Z. (2021). JBI Manual for Evidence Synthesis. https://jbi-global-wiki.refined.site/space/MANUAL.

[17] Higgins J.P.T., Thomas J., Chandler J., Cumpston M., Li T., Page M.J., Welch V.A. (2019). Cochrane Handbook for Systematic Reviews of Interventions. https://jhu.pure.elsevier.com/en/publications/cochrane-handbook-for-systematic-reviews-of-interventions.

[18] Page M.J., McKenzie J.E., Bossuyt P.M., Boutron I., Hoffmann T.C., Mulrow C.D., Shamseer L., Tetzlaff J.M., Akl E.A., Brennnan S.E. (2021). The PRISMA 2020 statement: An updated guideline for reporting systematic reviews. BMJ.

[19] Naficy A.B., Abu-Elyazeed R., Holmes J.L., Rao M.R., Savarino S.J., Kim Y., Wierzba T.F., Peruski L., Lee Y.J., Gentsch J.R. (1999). Epidemiology of rotavirus diarrhea in Egyptian children and implications for disease control. Am. J. Epidemiol..

[20] Allayeh A.K., El Baz R.M., Saeed N.M., Osman M.E.S. (2018). Detection and Genotyping of Viral Gastroenteritis in Hospitalized Children Below Five Years Old in Cairo, Egypt. Arch. Pediatr. Infect. Dis..

[21] Saudy N., Elshabrawy W.O., Megahed A., Foad M.F., Mohamed A.F. (2017). Genotyping and Clinicoepidemiological Characterization of Rotavirus Acute Gastroenteritis in Egyptian Children. Polish J. Microbiol..

[22] Elnady H., Abdelsamie O., Sallam S., Sherif L., Kholoussi N., Khoulousi S., Ali M. (2016). Genotyping of rota virus causing gastroenteritis in Egyptian children. Res. J. Pharm. Biol. Chem. Sci..

[23] Matson D.O., Abdel-Messih I.A., Schlett C.D., Bok K., Wienkopff T., Wierzba T.F., Sanders J.W., Frenck J.R.W. (2010). Rotavirus Genotypes among Hospitalized Children in Egypt, 2000–2002. J. Infect. Dis..

[24] Ahmed S.F., Mansour A.M., Klena J.D., Husain T.S., Hassan K.A., Mohamed F., Steele D. (2014). Rotavirus Genotypes Associated with Acute Diarrhea in Egyptian Infants. Pediatr. Infect. Dis. J..

[25] Benhafid M., Youbi M., Klena J.D., Gentsch J.R., Teleb N., Widdowson M., El Aouad R. (2009). Epidemiology of Rotavirus Gastroenteritis among Children <5 Years of Age in Morocco during 1 Year of Sentinel Hospital Surveillance, June 2006–May 2007. J. Infect. Dis..

[26] Benhafid M., Elomari N., Elqazoui M., Meryem A.I., Rguig A., Filali-Maltouf A., Elaouad R. (2013). Diversity of rotavirus strains circulating in children under 5 years of age admitted to hospital for acute gastroenteritis in Morocco, June 2006 to May 2009. J. Med. Virol..

[27] El Qazoui M., Oumzil H., Baassi L., El Omari N., Sadki K., Amzazi S., Benhafid M., El Aouad R. (2014). Rotavirus and norovirus infections among acute gastroenteritis children in Morocco. BMC Infect. Dis..

[28] Chouikha A., Fredj M.B.H., Fodha I., Mathlouthi I., Ardhaoui M., Teleb N., Brini I., Messaadi F., Mastouri M., Sfar T. (2011). Évolution des souches de Rotavirus du groupe A en circulation en Tunisie sur une période de trois ans (2005–2007). Pathol. Biol..

[29] Trabelsi A., Fodha I., Chouikha A., Fredj M.B.H., Mastouri M., Ben Abdelaziz A., Sfar T., Essoussi A.S., Jaoua S., Steele A.D. (2010). Rotavirus Strain Diversity in the Centre Coast of Tunisia from 2000 through 2003. J. Infect. Dis..

[30] Soltani M., Bouanene I., Trabelsi A., Harbi A., Hachicha M., Amri F., Boussnina S., Gueddiche M.N., Sfar M.T., Teleb N. (2012). Épidémiologie des gastroentérites à rotavirus chez les enfants âgés de moins de cinq ans en Tunisie—Résultats de la surveillance sentinelle hospitalière 2009 à 2011. Rev. Epidemiol. Sante Publique.

[31] Chouikha A., Fodha I., Noomen S., Bouzid L., Mastouri M., Peenze I., De Beer M., Dewar J., Geyer A., Sfar T. (2007). Group A rotavirus strains circulating in the eastern center of Tunisia during a ten-year period (1995–2004). J. Med. Virol..

[32] Moussa A., Fredj M.B.H., Fodha I., BenHamida-Rebaï M., Kacem S., Argoubi A., Bennour H., Boujaafar N., Trabelsi A. (2016). Distribution of rotavirus VP7 and VP4 genotypes circulating in Tunisia from 2009 to 2014: Emergence of the genotype G12. J. Med. Microbiol..

[33] Bennour H., Fodha I., Bouazizi A., Ben Hamida-Rebaï M., Jerbi A., Fredj M.B.H., Lakhal S., Dhiflaoui A., Abdelberi S., Abbassi F. (2019). Molecular characterization of group A rotavirus among children aged under 5 years in Tunisia, 2015–2017. J. Med. Microbiol..

[34] Agbla J.M., Esona M.D., Agbankpe A.J., Capo-Chichi A., Gautam R., Dougnon T.V., Razack O., Bowen M.D., Bankole H.S. (2020). Molecular characteristics of rotavirus genotypes circulating in the south of Benin, 2016–2018. BMC Res. Notes.

[35] Steele A.D., Page N., de Beer M., Sawadogo S. (2010). Antigenic and Molecular Characterization of Unusual Rotavirus Strains in Burkina Faso in 1999. J. Infect. Dis..

[36] Bonkoungou I.J., Damanka S., Sanou I., Tiendrébéogo F., Coulibaly S.O., Bon F., Haukka K., Traoré A.S., Barro N., Armah G.E. (2011). Genotype diversity of group A rotavirus strains in children with acute diarrhea in urban Burkina Faso, 2008–2010. J. Med. Virol..

[37] Rönnelid Y., Bonkoungou I.J.O., Ouedraogo N., Barro N., Svensson L., Nordgren J. (2020). Norovirus and rotavirus in children hospitalised with diarrhoea after rotavirus vaccine introduction in Burkina Faso. Epidemiol. Infect..

[38] Nordgren J., Bonkoungou I.J.O., Nitiema L.W., Sharma S., Ouermi D., Simpore J., Barro N., Svensson L. (2012). Rotavirus in diarrheal children in rural Burkina Faso: High prevalence of genotype G6P[6]. Infect. Genet. Evol..

[39] Bonkoungou I.J.O., Ouédraogo N., Tamini L., Teguera R.K., Yaméogo P., Drabo M.K., Medah I., Barro N., Sharma S., Svensson L. (2018). Rotavirus and norovirus in children with severe diarrhea in Burkina Faso before rotavirus vaccine introduction. J. Med. Virol..

[40] Asmah R.H., Green J., Armah G.E., Gallimore C.I., Gray J.J., Iturriza-Gόmara M., Anto F., Oduro A., Binka F.N., Brown D.W.G. (2001). Rotavirus G and P Genotypes in Rural Ghana. J. Clin. Microbiol..

[41] Armah G.E., Pager C.T., Asmah R.H., Anto F.R., Oduro A.R., Binka F., Steele D. (2001). Prevalence of unusual human rotavirus strains in Ghanaian children. J. Med. Virol..

[42] Binka F.N., Anto F.K., Oduro A.R., Awini E.A., Nazzar A.K., Armah G.E., Asmah R.H., Hall A.J., Cutts F., Alexander N. (2003). Incidence and risk factors of paediatric rotavirus diarrhoea in northern Ghana. Trop. Med. Int. Health.

[43] Enweronu-Laryea C.C., Sagoe K.W., Damanka S., Lartey B., Armah G. (2013). E Rotavirus genotypes associated with childhood severe acute diarrhoea in southern Ghana: A cross-sectional study. Virol. J..

[44] Lartey B.L., Damanka S., Dennis F.E., Enweronu-Laryea C.C., Addo-Yobo E., Ansong D., Kwarteng-Owusu S., Sagoe K.W., Mwenda J.M., Diamenu S.K. (2018). Rotavirus strain distribution in Ghana pre- and post- rotavirus vaccine introduction. Vaccine.

[45] Letsa V., Damanka S., Dennis F., Lartey B., Armah G.E., Betrapally N., Gautam R., Esona M.D., Bowen M.D., Quaye O. (2019). Distribution of rotavirus genotypes in the postvaccine introduction era in Ashaiman, Greater Accra Region, Ghana, 2014–2016. J. Med. Virol..

[46] Damanka S., Adiku T.K., Armah G.E., Rodrigues O., Donkor E.S., Nortey D., Asmah R. (2016). Rotavirus Infection in Children with Diarrhea at Korle-Bu Teaching Hospital, Ghana. Jpn. J. Infect. Dis..

[47] Nielsen N.M., Eugen-Olsen J., Aaby P., Mølbak K., Rodrigues A., Fischer T.K. (2005). Characterisation of rotavirus strains among hospi-talised and non-hospitalised children in Guinea-Bissau, 2002: A high frequency of mixed infections with serotype G8. J. Clin. Virol..

[48] Boni-Cisse C., Meite S., Mlan A.B., Zaba F., N’guessan R., Lepri N.A., Lartey B. (2018). Genotypic characterization of rotavirus in children under 5 years circulating in Côte D’Ivoire from 2010 to 2013. Virol. J..

[49] Page A.L., Jusot V., Mamaty A.A., Adamou L., Kaplon J., Pothier P., Djibo A., Manzo M.L., Toure B., Langendorf C. (2014). Rotavirus Surveillance in Urban and Rural Areas of Niger, April 2010–March 2012. Emerg. Infect. Dis..

[50] Audu R., Omilabu S.A., de Beer M., Peenze I., Steele A.D. (2002). Diversity of human rotavirus VP6, VP7, and VP4 in Lagos State, Nigeria. J. Health Popul. Nutr..

[51] Ianiro G., Delogu R., Baba M., Oderinde B.S., Dawurung J., Ruggeri F.M., Fiore L. (2015). Molecular characterization of group A rotavirus strains detected in children with diarrhea admitted to Nigerian hospitals in 2013. Arch. Virol..

[52] Ayolabi C.I. (2016). Genetic diversity of rotavirus strains in children with diarrhea in Lagos, Nigeria. Asian Pac. J. Trop. Dis..

[53] Uzoma E.B., Chukwubuikem C., Omoyibo E., Tagbo O. (2016). Rota virus genotypes and the clinical severity of Diarrhoea among children under 5 years of age. Niger. Postgrad. Med. J..

[54] Japhet M.O., Famurewa O., Iturriza-Gomara M., Adesina O.A., Opaleye O.O., Niendorf S., Bock C.T., Marques A.M. (2017). Group A rotaviruses circulating prior to a national immunization programme in Nigeria: Clinical manifestations, high G12P[8] frequency, intra-genotypic divergence of VP4 and VP7. J. Med. Virol..

[55] Amadu D.O., Abdullahi I.N., Emeribe A.U., Musa P.O., Olayem L., Yunusa T., Okechukwu C.E., Salami M.O. (2019). Molecular characterization of rotavirus genotype-A in children with acute diarrhea attending a tertiary hospital in Ilorin, Nigeria. Int. J. Health Allied Sci..

[56] Jere K.C., Sawyerr T., Seheri L.M., Peenze I., Page N.A., Geyer A., Steele A.D. (2011). A first report on the characterization of rotavirus strains in Sierra Leone. J. Med. Virol..

[57] Armah G.E., Steele A.D., Esona M.D., Akran V.A., Nimzing L., Pennap G. (2010). Diversity of Rotavirus Strains Circulating in West Africa from 1996 to 2000. J. Infect. Dis..

[58] Esona M.D., Armah G.E., Steele A.D. (2010). Rotavirus VP4 and VP7 Genotypes Circulating in Cameroon: Identification of Unusual Types. J. Infect. Dis..

[59] Boula A., Waku-Kouomou D., Njiki Kinkela M., Esona M.D., Kemajou G., Mekontso D., Seheri M., Ndze V.N., Emah I., Ela S. (2014). Molecular surveillance of rotavirus strains circulating in Yaoundé, Cameroon, September 2007–December 2012. Infect. Genet. Evol..

[60] Ndze V.N., Papp H., Achidi E.A., Gonsu K.H., László B., Farkas S., Kisfali P., Melegh B., Esona M.D., Bowen M.D. (2013). One year survey of human rotavirus strains suggests the emergence of genotype G12 in Cameroon. J. Med. Virol..

[61] Banga-Mingo V., Waku-Kouomou D., Gody J.C., Esona M.D., Yetimbi J.F., Mbary-Daba R., Dahl B.A., Dimanche L., Koyazegbe T.D., Tricou V. (2014). Molecular surveillance of rotavirus infection in Bangui, Central African Republic, October 2011–September 2013. Infect. Genet. Evol..

[62] Moure U.A.E., Banga-Mingo V., Gody J.C., Mwenda J.M., Fandema J., Waku-Kouomou D., Manengu C., Koyazegbe T.D., Esona M.D., Bowen M.D. (2018). Emergence of G12 and G9 rotavirus genotypes in the Central African Republic, January 2014 to February 2016. BMC Res. Notes.

[63] Mayindou G., Ngokana B., Sidibé A., Moundélé V., Koukouikila-Koussounda F., Christevy Vouvoungui J., Kwedi Nolna S., Velavan T.P., Ntoumi F. (2016). Molecular epidemiology and surveillance of circulating rotavirus and adenovirus in Congolese children with gastroenteritis. J. Med. Virol..

[64] Kabue J.P., Peenze I., de Beer M., Esona M.D., Lunfungula C., Biamungu M., Simba T.R., Tamfum J.J.M., Steele A.D. (2010). Characterization of Human Rotavirus Recovered from Children with Acute Diarrhea in Kinshasa, Democratic Republic of Congo. J. Infect. Dis..

[65] Pukuta E.S., Esona M.D., Nkongolo A., Seheri M., Makasi M., Nyembwe M., Mondonge V., Dahl B.A., Mphahlele M.J., Cavallaro K. (2014). Molecular Surveillance of Rotavirus Infection in the Democratic Republic of the Congo August 2009 to June 2012. Pediatr. Infect. Dis. J..

[66] Istrate C., Sharma S., Nordgren J., Videira e Castro S., Lopes Â., Piedade J., Zaky A., Lima A., Neves E., Veiga J. (2015). High rate of detection of G8P[6] rotavirus in children with acute gastroenteritis in São Tomé and Príncipe. Arch. Virol..

[67] Abebe A., Teka T., Kassa T.B., Seheri M., Beyene B.M., Teshome B., Kebede F., Habtamu A., Maake L.M., Kassahun A.M. (2014). Hospital-based Surveillance for Rotavirus Gastroenteritis in Children Younger Than 5 Years of Age in Ethiopia. Pediatr. Infect. Dis. J..

[68] Gelaw A., Pietsch C., Liebert U.G. (2018). Molecular epidemiology of rotaviruses in Northwest Ethiopia after national vaccine introduction. Infect. Genet. Evol..

[69] Nyangao J., Page N., Esona M., Peenze I., Gatheru Z., Tukei P., Steele A.D. (2010). Characterization of Human Rotavirus Strains from Children with Diarrhea in Nairobi and Kisumu, Kenya, between 2000 and 2002. J. Infect. Dis..

[70] Wandera E.A., Mohammad S., Bundi M., Nyangao J., Galata A., Kathiiko C., Odoyo E., Guyo S., Miring’u G., Komoto S. (2018). Impact of rotavirus vaccination on rotavirus hospitalisation rates among a resource-limited rural population in Mbita, Western Kenya. Trop. Med. Int. Health.

[71] Raini S., Nyangao J., Kombich J., Sang C., Gikonyo J., Ongus J., Odari E. (2015). Human Rotavirus Group a Serotypes Causing Gastroenteritis in Children Less Than 5 Years and HIV-Infected Adults in Viwandani Slum, Nairobi. Ethiop. J. Health Sci..

[72] Kiulia N.M., Nyaga M.M., Seheri M.L., Wolfaardt M., van Zyl W.B., Esona M.D., Irimu G., Inoti M., Gatinu B.W., Njenga P.K. (2014). Rotavirus G and P types circulating in the eastern region of Kenya: Predominance of G9 and emergence of G12 genotypes. Pediatr. Infect Dis. J..

[73] Wandera E.A., Mohammad S., Komoto S., Maeno Y., Nyangao J., Ide T., Kathiiko C., Odoyo E., Tsuji T., Taniguchi K. (2016). Molecular epidemiology of rotavirus gastroenteritis in Central Kenya before vaccine introduction, 2009-2014. J. Med. Virol..

[74] Cunliffe N.A., Gondwe J.S., Broadhead R.L., Molyneux M.E., Woods P.A., Bresee J.S., Glass R.I., Gentsch J.R., Hart C.A. (1999). Rotavirus G and P types in children with acute diarrhea in Blantyre, Malawi, from 1997 to 1998: Predominance of novel P[6]G8 strains. J. Med. Virol..

[75] Cunliffe N.A., Gondwe J.S., Graham S.M., Thindwa B.D.M., Dove W., Broadhead R.L., Molyneux M.E., Hart C.A. (2001). Rotavirus Strain Diversity in Blantyre, Malawi, from 1997 to 1999. J. Clin. Microbiol..

[76] Cunliffe N.A., Ngwira B.M., Dove W., Thindwa B.D.M., Turner A.M., Broadhead R.L., Molyneux M.E., Hart C.A. (2010). Epidemiology of Rotavirus Infection in Children in Blantyre, Malawi, 1997–2007. J. Infect. Dis..

[77] Turner A., Ngwira B., Witte D., Mwapasa M., Dove W., Cunliffe N. (2013). Surveillance of rotavirus gastro-enteritis in children in Blantyre, Malawi. Ann. Trop. Paediatr..

[78] João E.D., Munlela B., Chissaque A., Chilaúle J., Langa J., Augusto O., Boene S.S., Anapakala E., Sambo J., Guimarães E. (2020). Molecular Epidemiology of Rotavirus A Strains Pre- and Post-Vaccine (Rotarix®) Introduction in Mozambique, 2012–2019: Emergence of Genotypes G3P[4] and G3P[8]. Pathogens.

[79] Chissaque A., Bauhofer A.F.L., Cossa-Moiane I., Sitoe E., Munlela B., João E.D., Langa J.S., Chilaúle J.J., Boene S.S., Cassocera M. (2021). Rotavirus A infection in pre- and post-vaccine period: Risk factors, genotypes distribution by vaccination status and age of children in Nampula Province, Northern Mozambique (2015–2019). PLoS ONE.

[80] Manjate F., João E.D., Chirinda P., Garrine M., Vubil D., Nobela N., Kotloff K., Nataro J.P., Nhampossa T., Acácio S. (2022). Molecular Epidemiology of Rotavirus Strains in Symptomatic and Asymptomatic Children in Manhiça District, Southern Mozambique 2008–2019. Viruses.

[81] Hokororo A., Kidenya B.R., Seni J., Mapaseka S., Mphahlele J., Mshana S.E. (2014). Predominance of Rotavirus G1[P8] Genotype among Under-Five Children with Gastroenteritis in Mwanza, Tanzania. J. Trop. Pediatr..

[82] Odiit A.M.M., Mulindwa A.B., Nalumansi E.B., Mphahlele M.J., Seheri L.M., Mwenda J.M., Kisakye A. (2014). Rotavirus Prevalence and Genotypes Among Children Younger Than 5 Years With Acute Diarrhea at Mulago National Referral Hospital, Kampala, Uganda. Pediatr. Infect. Dis. J..

[83] Bwogi J., Malamba S., Kigozi B., Namuwulya P., Tushabe P., Kiguli S., Byarugaba D.K., Desselberger U., Iturriza-Gomara M., Karamagi C. (2016). The epidemiology of rotavirus disease in under-five-year-old children hospitalized with acute diarrhea in central Uganda, 2012–2013. Arch. Virol..

[84] Simwaka J., Seheri M., Mulundu G., Kaonga P., Mwenda J.M., Chilengi R., Mpabalwani E., Munsaka S. (2021). Rotavirus breakthrough infections responsible for gastroenteritis in vaccinated infants who presented with acute diarrhoea at University Teaching Hospitals, Children’s Hospital in 2016, in Lusaka Zambia. PLoS ONE.

[85] Mukaratirwa A., Berejena C., Nziramasanga P., Ticklay I., Gonah A., Nathoo K., Manangazira P., Mangwanya D., Marembo J., Mwenda J.M. (2018). Distribution of rotavirus genotypes associated with acute diarrhoea in Zimbabwean children less than five years old before and after rotavirus vaccine introduction. Vaccine.

[86] Mukaratirwa A., Berejena C., Nziramasanga P., Shonhai A., Mamvura T.S., Chibukira P., Mucheuki I., Mangwanya D., Kamupota M., Manangazira P. (2014). Epidemiologic and genotypic characteristics of rotavirus strains detected in children less than 5 years of age with gastroenteritis treated at 3 pediatric hospitals in Zimbabwe during 2008–2011. Pediatr. Infect. Dis. J..

[87] Mokomane M., Esona M.D., Bowen M.D., Tate J.E., Steenhoff A.P., Lechiile K., Gaseitsiwe S., Seheri L.M., Magagula N.B., Weldegebriel G. (2019). Diversity of Rotavirus Strains Circulating in Botswana before and after introduction of the Monovalent Rotavirus Vaccine. Vaccine.

[88] Page N., Pager C., Steele A.D. (2010). Characterization of Rotavirus Strains Detected in Windhoek, Namibia during 1998–1999. J. Infect. Dis..

[89] Seheri L.M., Page N., Dewar J.B., Geyer A., Nemarude A.L., Bos P., Esona M., Steele A.D. (2010). Characterization and Molecular Epidemiology of Rotavirus Strains Recovered in Northern Pretoria, South Africa during 2003–2006. J. Infect. Dis..

[90] Sanchez-Padilla E., Grais R.F., Guerin P.J., Steele A.D., Burny M.E., Luquero F.J. (2009). Burden of disease and circulating serotypes of rotavirus infection in sub-Saharan Africa: Systematic review and meta-analysis. Lancet Infect. Dis..

[91] Seheri M., Nemarude L.M., Peenze I.M., Netshifhefhe L.M., Nyaga M.M.M., Ngobeni H.G.M., Maphalala G.M., Maake L.L.M., Steele A.D., Mwenda J.M. (2014). Update of Rotavirus Strains Circulating in Africa From 2007 Through 2011. Pediatr. Infect. Dis. J..

[92] Santos N., Hoshino Y. (2004). Global distribution of rotavirus serotypes/genotypes and its implication for the development and implementation of an effective rotavirus vaccine. Rev. Med. Virol..

[93] Iturriza-Gómara M., Kang G., Gray J. (2004). Rotavirus genotyping: Keeping up with an evolving population of human rotaviruses. J. Clin. Virol..

[94] Dennehy P.H. (2008). Rotavirus Vaccines: An Overview. Clin. Microbiol. Rev..

[95] Burnett E., Jonesteller C.L., Tate J.E., Yen C., Parashar U.D. (2017). Global impact of rotavirus vaccination on childhood hospitalizations and mortality from diarrhea. J. Infect. Dis..

[96] Steele A.D., Armah G.E., Mwenda J.M., Kirkwood C.D. (2023). The Full Impact of Rotavirus Vaccines in Africa Has Yet to Be Realized. Clin. Infect. Dis..

[97] Hoxie I., Dennehy J.J. (2021). Rotavirus A Genome Segments Show Distinct Segregation and Codon Usage Patterns. Viruses.

