# Supplementary material for: Genetic Profile of Rotavirus Type A in Children under 5 Years Old in Africa: A Systematic Review of Prevalence
Source: Viruses. 2024 Feb 3;16(2):243. doi: 10.3390/v16020243 (PMC10893345; doi:10.3390/v16020243)
Supplement: Supplementary file 1 [file viruses-16-00243-s001.zip › viruses-2667497-supplementary/viruses-2667497-supplementary.pdf]

Supplemental Material

Table S1. Search Strategies

Data Base	Strategy	Nº of registries (12/07/2022)
**PubMed**	**#1** Rotavirus[MH] OR “Rotavirus Infections”[MH] OR Rotaviruses[TIAB] OR Rotavirus[TIAB]<br><br>**#2** “Genetic Profile”[MH] OR “Genetic Variation”[MH] OR “Molecular Epidemiology”[MH] OR “genetic diversity”[TIAB] OR “genetic variation”[TIAB] OR “genetic profile”[TIAB] OR “genetic profiling”[TIAB] OR “molecular profile”[TIAB] OR “molecular profiling”[TIAB] OR “molecular epidemiology”[TIAB] OR “genetic epidemiology”[TIAB] OR “genomic characterization”[TIAB] OR “genotypic characteristics”[TIAB] OR “molecular characterization”[TIAB] OR “molecular surveillance”[TIAB] OR strain*[TIAB] OR genotype*[TIAB] OR serotype*[TIAB]<br><br>**#3** Infant[MH] OR Child[MH] OR infant*[TIAB] OR child*[TIAB] OR pediatric*[TIAB] OR neonate*[TIAB] OR newborn*[TIAB]<br><br>**#4** Africa[MH] OR Africa*[TIAB] OR Afrique[TIAB] OR Argel*[TIAB] OR Algeri*[TIAB] OR Angola[TIAB] OR Benim[TIAB] OR Botswana[TIAB] OR Burkina[TIAB] OR Burquina[TIAB] OR Burundi[TIAB] OR Camarões[TIAB] OR Cameroon[TIAB] OR Cameroun[TIAB] OR “Cabo Verde”[TIAB] OR “Cape Verde”[TIAB] OR “Cap Vert”[TIAB] OR Chad*[TIAB] OR Tchad[TIAB] OR Comores[TIAB] OR Camoros[TIAB] OR Congo[TIAB] OR Ivory[TIAB] OR Ivoire[TIAB] OR Djibouti[TIAB] OR Egito[TIAB] OR Egypt*[TIAB] OR Guine*[TIAB] OR Eritre*[TIAB] OR Erythrée[TIAB] OR Etiópia[TIAB] OR Ethiopi*[TIAB] OR Gabão[TIAB] OR Gabon[TIAB] OR Gambia[TIAB] OR Gana[TIAB] OR Ghana[TIAB] OR Gambie[TIAB] OR Quênia[TIAB] OR Kenya[TIAB] OR Lesoto[TIAB] OR Lesotho[TIAB] OR Liberia[TIAB] OR Libia[TIAB] OR Liby*[TIAB] OR Madagascar[TIAB] OR Malawi[TIAB] OR Mali[TIAB] OR Mauritani*[TIAB] OR Mauricias[TIAB] OR Mauritius[TIAB] OR Maurice[TIAB] OR Marrocos[TIAB] OR Maroc*[TIAB] OR Moçambique[TIAB] OR Mozambique[TIAB] OR Namibi*[TIAB] OR Niger*[TIAB] OR Ruanda[TIAB] OR Rwanda[TIAB] OR “Sao Tome”[TIAB] OR Senegal[TIAB] OR Seychelles[TIAB] OR “Serra Leoa”[TIAB] OR Leone[TIAB] OR Somali*[TIAB] OR Sudan[TIAB] OR Sudão[TIAB] OR Soudan[TIAB] OR Suazilândia[TIAB] OR Swaziland OR Tanzani*[TIAB] OR Togo[TIAB] OR Tunisi*[TIAB] OR Uganda[TIAB] OR Ouganda[TIAB] OR Zambi*[TIAB] OR Zimbab*[TIAB]	472

Supplemental Material

Table S1. Search Strategies

Data Base	Strategy	Nº of registries (12/07/2022)
**Scopus**	**#1** TITLE-ABS-KEY(Rotaviruses OR Rotavirus)<br><br>**#2** TITLE-ABS(“genetic diversity” OR “genetic variation” OR “genetic profile” OR “genetic profiling” OR “molecular profile” OR “molecular profiling” OR “molecular epidemiology” OR “genetic epidemiology” OR “genomic characterization” OR “genotypic characteristics” OR “molecular characterization” OR “molecular surveillance” OR strain* OR genotype* OR serotype*)<br><br>**#3** TITLE-ABS(infant* OR child* OR pediatric* OR neonate* OR newborn*)<br><br>**#4** TITLE-ABS-KEY(Africa* OR Afrique OR Argel* OR Algeri* OR Angola OR Benim OR Botswana OR Burkina OR Burquina OR Burundi OR Camarões OR Cameroon OR Cameroun OR “Cabo Verde” OR “Cape Verde” OR “Cap Vert” OR Chad* OR Tchad OR Comores OR Camoros OR Congo OR Ivory OR Ivoire OR Djibouti OR Egito OR Egypt* OR Guine* OR Eritre* OR Erythrée OR Etiópia OR Ethiopi* OR Gabão OR Gabon OR Gambia OR Gana OR Ghana OR Gambie OR Quênia OR Kenya OR Lesoto OR Lesotho OR Liberia OR Libia OR Liby* OR Madagascar OR Malawi OR Mali OR Mauritani* OR Mauricias OR Mauritius OR Maurice OR Marrocos OR Maroc* OR Moçambique OR Mozambique OR Namibi* OR Niger* OR Ruanda OR Rwanda OR “Sao Tome” OR Senegal OR Seychelles OR “Serra Leoa” OR Leone OR Somali* OR Sudan OR Sudão OR Soudan OR Suazilândia OR Swaziland OR Tanzani* OR Togo OR Tunisi* OR Uganda OR Ouganda OR Zambi* OR Zimbab*)	467

Supplemental Material

Table S1. Search Strategies

Data Base	Strategy	Nº of registries (12/07/2022)
**Web of Science**	Rotaviruses OR Rotavirus (Tópico) and “genetic diversity” OR “genetic variation” OR “genetic profile” OR “genetic profiling” OR “molecular profile” OR “molecular profiling” OR “molecular epidemiology” OR “genetic epidemiology” OR “genomic characterization” OR “genotypic characteristics” OR “molecular characterization” OR “molecular surveillance” OR strain* OR genotype* OR serotype* (Tópico) and infant* OR child* OR pediatric* OR neonate* OR newborn* (Tópico) and Africa* OR Afrique OR Argel* OR Algeri* OR Angola OR benin OR Botswana OR Burkina OR bourquina OR Burundi OR Camarões OR Cameroon OR Cameroun OR “Cabo Verde” OR “Cape Verde” OR “Cap Vert” OR Chad* OR Tchad OR Comores OR comoros OR Congo OR Ivory OR Ivoire OR Djibouti OR exito OR Egypt* OR Guine* OR Eritre* OR Erythrée OR Etiópia OR Ethiopi* OR Gabão OR Gabon OR Gambia OR Gana OR Ghana OR gambiae OR Quênia OR Kenya OR lesotho OR Lesotho OR Liberia OR libya OR Liby* OR Madagascar OR Malawi OR Mali OR Mauritani* OR mauricius OR Mauritius OR Maurice OR marroco OR Maroc* OR Moçambique OR Mozambique OR Namibi* OR Niger* OR Ruanda OR Rwanda OR “Sao Tome” OR Senegal OR Seychelles OR “Serra Leoa” OR Leone OR Somali* OR Sudan OR Sudão OR Soudan OR Suazilândia OR Swaziland OR Tanzani* OR Togo OR Tunisi* OR Uganda OR organda OR Zambi* OR Zimbab* (Tópico) https://www.webofscience.com/wos/woscc/summary/27c35d58-b93f-4119-8a38-0d31dde129d4-42d32afd/relevance/1	524
**Scielo**	(rotavirus) AND (Africa OR Algeria OR Angola OR Benim OR Botswana OR Burkina OR Burundi OR Camarões OR Cameroon OR Cameroun OR “Cabo Verde” OR Chade OR Tchad OR Comores OR Camoros OR Congo OR Ivory OR Ivoire OR Djibouti OR Egito OR Egypt OR Guinea OR Etiópia OR Ethiopia OR Gabão OR Gabon OR Gambia OR Gana OR Ghana OR Gambie OR Quênia OR Kenya OR Lesoto OR Lesotho OR Liberia OR Libia OR Libya OR Madagascar OR Malawi OR Mali OR Mauritania OR Mauricias OR Mauritius OR Maurice OR Marrocos OR Marocco OR Moçambique OR Mozambique OR Namibia OR Nigeria OR Ruanda OR Rwanda OR “Sao Tome” OR Senegal OR Seychelles OR “Serra Leoa” OR Leone OR Somalia OR Sudan OR Sudão OR Soudan OR Suazilândia OR Swaziland OR Tanzania OR Togo OR Tunisia OR Uganda OR Ouganda OR Zambia OR Zimbab*)	12

Supplemental Material

Table S2. Characteristics of the Included Studies

N	Author (Data of Publication)	Country	Region of Africa	Year (s) of sample collections	Study Period (months)	Study Design	Study Setting	Nº of genotyped Samples	Genotyping Method	Vaccine Introduction Period*	List of genotypes found in the study
393	Naficy AB, et al. (1999)	Egypt	North	1995-1996	13	cohort	out	46	RT-PCR	Before	G1, G2, G8; P[4],P[8],P[14]; G1P[8], G2P[4], G8P[4], Mix G-P.
512	Allayeh AK, et al. (2018)	Egypt	North	2015-2016	12	surveillance hospital based	in	37	RT-PCR	Before	G1, G3, G9; P[4], P[6], P[8]; G1P[4], G1P[6], G1P[8], G3P[4], G3P[8], G9P[8], Mix G-P.
127	Saudy N, et al. (2017)	Egypt	North	2010-2012	18	cross-sectional -hospital (1) based	in	45	multiplex RT-PCR	Before	G1, G3, G9; P[4], P[8]; G1P[4], G1P[6], G1P[8], G3P[4], G3P[8], G9P[6],G9P[8] Mix G-P.
613	Elnady HG, et al. (2016)	Egypt	North	2012-2012	5	cross-sectional - hospital (4) based	in and out	53	RT-PCR	Before	G1, G2, G3, G4, G9; P[4],P[6], P[8]; G1P[8], G2P[4], G3P[8], G4P[8],G9P[6], G9P[8], G12P[6]; Mix G-P.
291	Matson DO, et al. (2010)	Egypt	North	2000 - 2002	24	surveillance hospital based	in	243	nested RT-PCR	Before	G1, G2, G3, G4,G9; P[4],P[6], P[8]; G1P[4], G1P[6], G1P[8], G2P[4], G2P[8], G3P[4], G4P[8], G9P[6], G9P[8], Mix G-P.
209	Ahmed SF, et al. (2014)	Egypt	North	2004-2007	40	cohort	out	164	nested, multiplex RT-PCR	Before	G1, G2, G9; P[4], P[6], P[8]; G1P[8], G2P[4], G2P[6], G2P[8], G3P[8], G9P[4], G9P[6], G9P[8] , G12 P[6], Mix P-G.
316	Benhafid M, et al. (2009)	Morocco	North	2006-2007	12	cross-sectional	in	134	RT-PCR	Before	G1, G2, G9; P[4], P[6], P[8]; G1P[6], G1P[8], G2P[4], G2P[6], G2P[8], G9P[8], Mix P-G.
241	Benhafid M, et al. (2013)	Morocco	North	2006-2009	36	cross-sectional	in	548	semi-nested, multiplex RT-PCR	Before	G1, G2, G3, G4, G9; P[4],P[6], P[8]; G1P[4], G1P[6], G1P[8], G2P[4], G2P[6], G2P[8], G3P[4], G3P[6], G3P[8], G4P[8], G9P[6], G9P[8]; Mix G-P.
196	El Qazoui M, et al. (2014)	Morocco	North	2011	6	Sentinel surveillance	in and out	89	multiplex RT-PCR	After	G1, G2, G4, G9; P[4], P[6], P[8] ; G1P[6], G1P[8], G2P[4], G2P[8], G4P[8], G9 P[8], Mix- G-P.
314	Chouikha A, et al. (2011)	Tunisia	North	2005-2007	36	surveillance hospital (8) based	in and out	323	semi-nested, multiplex RT-PCR	Before	G1, G2, G3, G4, G9; P[4], P[6], P[8], P[11]; G1P[6], G1P[8], G1P[11], G2P[4], G2P[6], G2P[8], G2P[11], G3P[4], G3P[6], G3P[8], G3P[11], G4P[6], G4P[8], G4P[11], G9P[6], G9P[8], G9P[11], Mix G-P.
292	Trabelsi A, et al. (2010)	Tunisia	North	2000 - 2003	45	cross-sectional	in and out	63	semi-nested multiplex RT-PCR	Before	G1, G2, G3, G4, G8; P[4], P[6], P[8], G1P[6], G1P[8], G2P[4], G3P[4], G3P[8], G4P[6], G4P[8], G6P[8], Mix- GP.
238	Soltani M, et al. (2012)	Tunisia	North	2009 - 2011	24	cross-sectional	in	188	semi-nested, multiplex RT-PCR	Before	G1, G2, G3, G4; P[4], P[6], P[8], P[9]; G1P[8], G2P[4], G3P[8], G4P[6], G4P[8], Mix G-P.

343	Chouikha A, et al. (2007)	Tunisia	North	2005-2007	36	surveillance	in and out	180	semi-nested, multiplex RT-PCR	Before	G1, G2, G3, G4, G8, G9; P[4], P[6], P[8]; G1P[4], G1P[6], G1P[8], G2P[4], G3P[4], G3P[6], G3P[8], G4P[4], G4P[6], G4P[8], G9P[8], Mix G-P.
151	Moussa A, et al. (2016)	Tunisia	North	2009-2014	60	cross-sectional	in and out	270	semi-nested RT-PCR	Before	G1, G2, G3, G4, G9, G12; P[4], P[6], P[8]; G1P[4], G1P[6], G1P[8], G2P[4], G2P[6], G2P[8], G3P[4], G3P[6], G3P[8], G4P[4], G4P[6], G4P[8], G9P[6], G9P[8], G12P[6], G12P[8], Mix G-P.
81	Bennour H, et al. (2019)	Tunisia	North	2015-2017	36	surveillance 3 - hosp	in and out	72	multiplex RT-PCR	Before	G1, G2, G3, G9, G12; P[4], P[6], P[8]; G1P[6], G1P[8], G2P[4], G2P[8], G3P[8], G9P[8], G12P[8], Mix G-P.
37	Agbla JM, et al (2020)	Benin	West	2016-2018	30	cross-sectional	out	186	multiplex RT-PCR	Before	G1, G2, G3, G9, G12; P[4], P[6], P[8]; G1P[6], G1P[8], G2P[4], G2P[6], G2P[8], G3P[6], G3P[8], G9P[4], G9P[8], G12P[6], G12P[8], Mix G-P.
294	Steele AD, et al. (2010)	Burkina Faso	West	1994	No	cross-sectional	out	36	RT-PCR	Before	G1, G2; P[6], P[8]
271	Bonkoungou IJ, et al. (2011)	Burkina Faso	West	2008 - 2010	16	cross-sectional	in and out	140	semi-nested multiplex RT-PCR	Before	G1, G2, G3, G4, G9, G10, G12; P[4], P[6], P[8], P[9]; G1P[6], G1P[8], G1P[9], G2P[4], G2P[6], G2P[8], G4P[6], G2P[8], G9P[6], G9P[8], G10P[6], G12P[6], Mix G-P.
40	Rönnelid Y, et al. (2020)	Burkina Faso	West	2015-2015	12	cross-sectional	in	20	multiplex RT-PCR	After	G1, G2, G12; P[4], P[6], P[8]; G1P[6], G1P[8], G2P[4], G12P[6], G12P[8]
243	Nordgren J, et al. (2012)	Burkina Faso	West	2010	2	cross-sectional	out	56	semi-nested, multiplex RT-PCR	Before	G1, G2, G6; G8, G9; P[4], P[6],P[8]; G1P[6], G1P[8], G2P[4], G2P[6], G6P[6], G8P[6], G9P[6], G9P[8]
260	Nordgren J, et al. (2012)	Burkina Faso	West	2009-2010	11	cross-sectional	out	100	semi-nested, multiplex RT-PCR	Before	G1, G2, G3, G6, G9; P[4], P[6], P[8], G1P[6], G1P[8], G2P[4], G3P[6], G6P[6], G9P[8], Mix G-P.
106	Bonkoungou IJO, et al. (2018)	Burkina Faso	West	2012-2013	12	surveillance hospital (1) based	in	67	RT-PCR	Before	G2, G6, G12; P[4], P[6], P[8]; G2P[4], G6P[6], G6P[8], G12P[6], G12P[8],Mix G-P.
385	Armah GE, et al. (2001)	Ghana	West	1999	4	cross-sectional	out	46	RT-PCR	Before	G2, G8; P[6], P[8]; G2P[6], G8P[6], Mix G-P.
382	Asmah RH, et al. (2001)	Ghana	West	1998	5	cross-sectional	out	50	semi-nested, multiplex RT-PCR	Before	G1, G2, G3; P[4], P[6], P[8]; G1P[6], G2P[6], G3P[4], G3P[6], G3P[8], Mix G-P.
367	Binka FN, et ai. (2003)	Ghana	West	1998-2020	24	cross-sectional	out	238	semi-nested PCR	Before	G1, G2, G3, G8, G9; P[4], P[6], P[8], P[10]; G1P[6], G1P[8], G2P[4], G2P[6], G2P[8], G3P[4], G3P[6], G3P[8], G8 P[6], G8P[8], G9P[6], G9P[8], G9P[10], Mix G-P.
219	Enweronu-Laryea CC, et al. (2013)	Ghana	West	2007-2011	42	cross-sectional	in	876	RT-PCR	Before	G1, G2, G3, G4, G8, G9, G10, G12; P[4], P[6], P[8]; G1P[4], G1P[6], G1P[8], G2P[4], G2P[6], G2P[8], G3P[4], G3P[6], G3P[8], G4P[4], G4P[6], G9P[6] , G9P[8], G10P[4], G10P[6], G12P[6], G12P[8]
112	Lartey BL, et al. (2018)	Ghana	West	2009-2016	96	surveillance	in	1363	RT-PCR	Before and After	Before: G1, G2, G3, G4, G6, G8, G9, G10, G12; P[4], P[6], P[8]; G1P[4], G1P[6], G1P[8], G2P[4], G2P[6], G2P[8], G3P[4], G3P[6], G3P[8], G4P[4], G4P[6], G4P[8], G6P[6], G8P[6], G9P[6], G9P[8], G10P[6], G12P[6], Mix G-P. After: G1, G2, G3, G4, G8, G9, G10, G12; P[4], P[6], P[8]; G1P[4], G1P[6], G1P[8], G2P[4], G2P[6], G2P[8], G3P[6], G3P[8], G4P[6], G4P[8], G8 P[8], G9 P[4], G9 P[6], G9 P[8], G10 P[4], G10 P[6], G10 P[8], G12P[4], G12P[6], G12P[8], Mix G-P.
79	Letsa V, et al. (2019)	Ghana	West	2014-2016	18	cross-sectional	out	136	semi-nested, multiplex RT-PCR	After	G1, G3, G9, G12; P[4], P[6], P[8], G1P[6], G1P[8], G3P[6], G3P[8], G9P[4], G9P[8], G10P[8], G12P[8]

166	Damanka S, at al. (2016)	Ghana	West	2004-2005	13	cross-sectional	in	70	RT-PCR	Before	G1, G2, G3, G9; P[4], P[6], P[8], P[9]; G1P[6], G1P[8], G1P[9], G2P[4], G2P[6], G2P[8], G3P[6], G9P[6], G9P[8]
356	Nielsen NM, et al. (2005)	Guinea-Bissau	West	2002	No	cross-sectional	in and out	104	multiplex RT-PCR	Before	G1, G2, G3, G8, G9; P[4], P[6], P[8]; G1P[6], G1P[8], G2P[4], G2P[6], G3P[6], G8P[4], G8 P[6], G8P[8], Mix G-P.
107	Boni-Cisse C, et al. (2018)	Ivory Coast	West	2010-2013	36	surveillance	in and out	186	multiplex RT-PCR	Before	G1, G2, G3; G9, G9, G12; P[4], P[6], P[8]; G1P[6], G1P[8], G2P[4], G2P[6], G3P[6], G3P[8], G8P[8], G9P[6], G12P[6], G12P[8]
200	Page AL, et al. (Jusot V, Mamaty AA, Adamou L, Kaplon J, Pothier P, Djibo A, Manzo ML, Toure B, Langendorf C, Collard JM, Grais RF	Niger	West	2010-2013	24	surveillance	out	449	RT-PCR	Before	G1, G2, G3, G6, G9, G12; P[4], P[6], P[8]; G1P[8], G2P[4], G2P[6] , G6P[6], G9P[8], G12P[8], Mix G-P.
376	Audu R, et al. (2002)	Nigeria	West	1996-1997	24	cross-sectional	in and out	23	RT-PCR	Before	G1, G3; P[4], P[6], P[8]; G1P[6]; G1P[8], G3P[8], Mix G-P
178	Ianiro G, et al. (2015)	Nigeria	West	2013	6	surveillance	in	66	nested RT-PCR	Before	G1, G2, G3, G12; P[4], P[6], P[8]; G1P[8], G2P[4], G3P[4], G3P[6], G3P[8], G12P[4], G12P[8], Mix G-P.
529	Ayolabi CI (2016)	Nigeria	West	2007-2008	12	cross-sectional	out	58	RT-PCR	Before	G1, G2, G3, G4,G9, G12;P[4], P[6], P[8]; G1P[6],G1P[8], G2P[4], G2P[6], G2P[8], G3P[6], G4P[4], G9P[6], G12P[8], Mix G-P.
155	Uzoma EB, et al. (2016)	Nigeria	West	2012-2013	12	cross-sectional	out	49	nested, multiplex RT-PCR	Before	G1, G3, G9, G12; P[4], P[6], P[8], G1P[6], G3P[6], G12P[8]
125	Japhet MO, et al. (2018)	Nigeria	West	2012-2013	17	cross-sectional	in and out	49	semi-nested, multiplex RT-PCR	Before	G2, G3, G9, G12; P[4], P[6], P[8]; G2P[4], G2P[6], G3P[6], G9P[4], G9P[8], G12P[8], Mix G-P.
1361	Amadu DO, et al. (2019)	Nigeria	West	2013-2014	16	surveillance hospital (1) based	in	25	multiplex RT-PCR	Before	G1, G2, G9, G10, G12; P[4], P[6], P[8]; G1P[6], G1P[8], G2P[6], G2P[8], G9P[4], G10P[6], G12P[4]
277	Jere KC, et al. (2011)	Sierra Leone	West	2005	6	cross-sectional	in	43	RT-PCR	Before	G2, G8; P[4], P[6], P[8]; G2P[4], G2P[6], G2P[8], G8P[4], G8P[6], Mix G-P.
287	Armah GE, et al. (2010)	Burkina Faso; Ivory Coast; Ghana, Nigeria, Cameroon.	West	1996-2000	45	surveillance 5 countries	in and out	925	RT-PCR	Before	
161	Esteves A, et al. (2016)	Angola	Central	2012-2013	17	cross-sectional	out	116	semi-nested, multiplex RT-PCR	Before	G1, G2, G8, G9, G12; P[4], P[6], P[8]; G1P[6], G1P[8],G2P[4], G8P[6], G9P[6], G12P[6].
135	Gasparinho C, et al. (2017)	Angola	Central	2012-2013	16	cross-sectional	out	72	semi-nested, multiplex RT-PCR	Before	G1, G2; P[4], P[6], P[8]; G1P[6], G1P[8], G2P[4]
297	Esona MD, et al. (2010)	Cameroon	Central	1999-2000	12	cross-sectional	out	89	RT-PCR	Before	G1, G2, G3, G4, G5, G8, G9, G10; P[4], P[6], P[8], P[9], P[10]; G1P[4], G1P[8], G1P[10], G2P[4], G2P[8], G3P[4], G3P[8], G3P[9], G4P[4], G4P[8], G5P[8], G8P[8], G9P[4], G9P[6], G9P[8], G10P[8], Mix G-P.
190	Boula A, et al. (2014)	Cameroon	Central	2007-2012	64	surveillance	in	898	semi-nested, multiplex RT-PCR	Before	G1, G2, G3, G4, G6, G8, G9, G12; P[4], P[6], P[8], P[9], P[14] ;G1P[4], G1P[6], G1P[8], G2P[4], G2P[6], G2P[8] , G3P[4], G3P[6], G3P[8], G4P[6], G4P[8], G6P[6], G6P[8], G8P[4], G8P[6], G8P[8], G9P[4], G9P[6], G9P[8], G12P[4], G12P[6], G12P[8], Mix G-P.
224	Ndze VN, et al. (2013)	Cameroon	Central	2010-2011	No	cross-sectional	out	135	RT-PCR	Before	G1, G2, G3, G8, G9, G12; P[4], P[6], P[8]; G1P[6], G2P[4], G2P[6], G3P[6], G3P[8], G8P[6], G9P[8], G12P[6], G12P[8], Mix G-P.
191	Banga-Mingo V, et al. (2014)	CAR	Central	2011-2013	24	cross-sectional	in	160	multiple RT-PCR	Before	G1, G2, G9, G12; P[4], P[6], P[8]; G1P[6], G1P[8], G2P[4], G2P[6], G2P[8], G9P[8], G12P[6], G12P[8].
113	Moure UAE, et al. (2018)	CAR	Central	2014-2016	26	surveillance hospital (1) based	in	100	semi-nested, multiplex RT-PCR	Before	G1, G2, G3, G9, G12; P[4], P[6], P[8]; G1P[6], G1P[8], G2P[4], G2P[6], G3P[6], G3P[8], G9P[6], G9P[8], G12P[6].

165	Mayindou G, et al. (2016)	Congo	Central	2012-2013	13	cross-sectional	in	219	RT-PCR	Before	G1, G2, G9, G10, G12; P[6], P[8]; G1P[6], G1P[8], G2P[6], G2P[8], G9P[8], G10P[8], G12P[6], Mix G-P.
299	Kabue JP, et al. (2010)	DRC	Central	2003-2005	12	cross-sectional	in	119	RT-PCR	Before	G1, G4, G8, G9; P[6], P[8]; G1P[6], G1P[8], G4P[6], G8P[6], G8P[8], G9P[6], Mix G-P.
201	Pukuta ES, et al. (2014)	DRC	Central	2009-2012	36	surveillance hospital (3) based	in	330	multiplex RT-PCR	Before	G1, G2, G3, G4, G6, G8, G9; P[4], P[6], P[8]; G1P[4], G1P[6], G1P[8], G2P[4], G2P[6], G2P[8], G3P[6],G4P[4], G4P[6], G6P[6], G6P[8], G8P[4], G8P[6], G8P[8], G9P[8], G12P[4], G12P[6], G12P[8], Mix G-P.
189	Istrate C, et al. (2015)	São Tome and Principe	Central	2011	5	surveillance hospital (6) based	out	83	semi-nested, multiplex RT-PCR	Before	G1, G8; P[6], P[8]; G1P[8], G8P[6], G8P[8].
213	Abebe A, et al. (2014)	Ethiopia	East	2007-2012	56	surveillance	in	215	semi-nested, multiplex RT-PCR	Before	G1, G2, G3, G9, G12; P[4], P[6], P[8]; G1P[6], G1P[8], G2P[4], G2P[6], G3P[6], G9P[6], G9P[8], G12P[6], G12P[8] .
96	Gelaw A, et al. (2018)	Ethiopia	East	2015-2016	6	cross-sectional	out	125	RT-PCR	After	G2, G3, G9, G12; P[4], P[6], P[8]; G2P[4], G3P[6], G3P[8], G9P[8], G12P[8].
300	Nyangao J, et al. (2010)	Kenya	East	2000-2002	36	cross-sectional	out	108	nested RT-PCR	Before	G1, G2, G3, G8, G9; P[4], P[6], P[8]; G1P[6], G1P[8], G2P[4], G2P[6], G2P[8], G3P[8], G8P[4], G8P[6], G9P[4], G9P[6], G9P[8], Mix G-P.
146	Wandera EA, et al. (2017)	Kenya	East	2009-2014	60	surveillance	in	429	semi-nested, multiplex RT-PCR	Before	G1, G2, G3, G4, G8, G9, G12; P[4], P[6], P[8]; G1P[4], G1P[6], G1P[8], G2P[4], G2P[8], G3P[4], G3P[6], G3P[8], G4P[4], G4P[8], G8P[4], G8P[6], G8P[8], G9P[4] , G9P[6], G9P[8], G12P[4], G12P[6], G12P[8], Mix G-P.
180	Raini SK, et al. (2015)	Kenya	East	2012-2013	12	cross-sectional	out	30	nested RT-PCR	Before	G1, G3, G9; P[6], P[8]; G1P[8], G3P[8], G9P[6], G9P[8].
206	Kiulia NM, et al. (2014)	Kenya	East	2009-2011	24	surveillance	in	157	RT-PCR	Before	G1, G2, G8, G9, G12; P[4], P[6], P[8]; G1P[8], GP[4], G8P[4], G8P[8], G9P[4], G9P[6], G9P[8], G12P[6], Mix G-P .
111	Wandera EA, et al. (2018)	Kenya	East	2011-2016	60	surveillance hospital based	in	61	semi-nested, multiplex RT-PCR	Before and After	Before: G1, G2, G3, G4, G8, G12; P[4], P[6], P[8]; G1P[4], G1P[8], G2P[4], G2P[6], G3P[6]. G4P[6], G4P[8], G8P[4], G8P[6], G8P[8], G12P[6], Mix G-P. After: G1, G2; G1P[8], G2P[4], G2P[6]
397	Cunliffe NA, et al. (1999)	Malawi	East	1997-1998	6	cross-sectional	in and out	100	semi-nested, multiplex RT-PCR	Before	G3, G4, G8, G9; P[4], P[6], P[8]; G3P[4], G3P[6], G3P[8], G4P[6], G4P[8], G8P[6], G9P[6], Mix G-P.
383	Cunliffe NA, et al. (2001)	Malawi	East	1997-1999	24	cross-sectional	in and out	414	multiplex RT-PCR	Before	G1, G3, G4, G8, G9; P[4], P[6], P[8]; G1P[6], G1P[8], G3P[4], G3P[6], G3P[8], G4P[6], G4P[8], G8P[6], G9P[6].
303	Cunliffe NA, et al. (2010)	Malawi	East	1997-1999	120	cross-sectional	out	1130	RT-PCR	Before	G1, G3, G4, G8, G9, G12; P[4], P[6], P[8]; G1P[4], G1P[6], G1P[8], G3P[4], G3P[6], G3P[8], G4P[6], G4P[8], G8P[4], G8P[6], G8P[8], G9P[6] , G9P[8], G12P[6], G12P[8], Mix G-P .
233	Turner A, et al. (2013)	Malawi	East	2008-2009	24	surveillance	in and out	220	semi-nested, multiplex RT-PCR	Before	G1, G2, G8, G9, G12; P[4], P[6], P[8]; G1P[4], G1P[6], G1P[8], G2P[4], G2P[6], G2P[8], G8P[4], G8P[6], G8P[8], G9P[6], G9P[8], G12P[6], G12P[8].
43	João ED, et al. (2020)	Mozambique	East	2015-2019	68	surveillance (5 sentinel sites)	in and out	650	RT-PCR	Before and After	Before: G1, G2, G9, G12; P[4], P[6], P[8]; G1P[8], G2P[4], G2P[6], G9P[8]. After: G1, G2, G3, G8, G9, G12; P[4], P[6], P[8], G1P[8], G3P[4], G3P[6], G3P[8], G8P[4], G9P[4], G9P[6], G2P[4], G2P[6], G9P[8], Mix G-P.
120	João ED, et al. (2018)	Mozambique	East	2012-2013	20	cross-sectional	in	157	semi-nested, multiplex RT-PCR	Before	G1, G2, G8, G9, G12; P[4], P[6], P[8]; G1P[8], G2P[4], G2P[8], G8P[4], G8P[8], G9P[8], G12P[4], G12P[6], G12P[8], Mix G-P .

18	Chissaque A, et al. (2021)	Mozambique	East	2015-2019	58	cross-sectional	out	152	RT-PCR	Before and After	Before: G1, G2, G9 ; P[6], P[8]; G1P[6], G1P[8], G2P[6], G9P[8]. After: G1, G2, G3, G9, G12; P[4], P[6], P[8]; G1P[8], G3P[4], G3P[8], G9P[4], G9P[6], G9P[8], G12P[8], Mix G-P.
8	Manjate F, et al. (2022)	Mozambique	East	2008-2012; 2016-2019	112	surveillance	in and out	291	semi-nested, multiplex RT-PCR	Before and After	Before: G1, G2, G3, G9, G12; P[4], P[6], P[8]; G1P[4], G1P[8], G2P[4], G2P[6], G3P[4], G3P[8], G9P[8], G12P[4], G12P[6], G12P[8]. After: G1, G2, G3, G9, G12; P[4], P[6], P[8], G1P[4], G1P[8], G2P[4], G2P[6], G3P[4], G3P[8], G9P[6], G9P[8], G12P[4], G12P[6], Mix G-P.
197	Hokororo A, et al. (2014)	Tanzania	East	2010-2012	30	cross-sectional	in	100	multiplex RT-PCR	Before	G1, G2, G8, G9; P[4], P[6], P[8]; G1P[6], G1P[8], G2P[4], G8P[4], G8P[6], G8P[8], Mix G-P.
212	Odiit A, et al. (2014)	Uganda	East	2006-2012	78	surveillance hospital based	in	354	semi-nested, RT-PCR	Before	G1, G2, G3, G8, G9, G12; P[4], P[6], P[8]; G1P[6], G1P[8], G2P[4], G3P[6], G8P[4], G8P[6], G8P[8], G9P[6] ,G9P[8], G12P[6].
162	Bwogi J, et al. (2016)	Uganda	East	2012-2013	13	cross-sectional	in	204	nested RT-PCR	Before	G1, G3, G9, G12; P[4], P[6], P[8]; G1P[8], G3P[4], G3P[6], G9P[4], G9P[6], GP[8], G12P[4], G12P[6], G12P[8], Mix G-P.
29	Simwaka J, et al. (2021)	Zambia	East	2016	no	surveillance	in	116	RT-PCR	After	G1, G2, G6, G9 ; P[4], P[6], P[8]; G1P[6], G1P[8], G2P[4], G2P[6], G6P[6], G8P[6], G9P[6], Mix- G-P.
109	Mukaratirwa A, at al. (2018)	Zimbabwe	East	2008-2016	96	surveillance hospital (3) based	in and out	1096	RT-PCR	Before and After	Before: G1, G2, G3, G4, G8, G9, G12; P[4], P[6], P[8]; G1P[4], G1P[6], G1P[8], G2P[4], G2P[6], G2P[8], G8P[4], G8P[6], G9P[4], G9P[6], G9P[8], G12P[4], G12P[6], G12P[8], Mix G-P. After: G1, G2, G3, G4, G8, G9, G12; P[4], P[6], P[8]; G1P[4], G1P[6], G1P[8], G2P[4], G2P[6], G2P[8], G3 P[4], G3 P[6], G8P[6], G9P[4], G9P[6], G9P[8], G12P[6], G12P[8], Mix G-P.
211	Mukaratirwa A, et al. (2014)	Zimbabwe	East	2008-2011	48	surveillance hospital based	in	127	RT-PCR	Before	G1, G2, G8, G9, G12; P[4], P[6], P[8]; G1P[6], G1P[8], G2P[4], G2P[6], G8P[4], G8P[6], G9P[4], G9P[6], G9P[8], G12P[4], G12P[6], G12P[8], Mix G-P.
74	Mokomane M, et al. (2019)	Botswana	South	2011-2018	91	surveillance	in	284	multiplex RT-PCR	Before and After	Before: G1, G2, G9, G12; P[4], P[6], P[8]; G1P[8], G2P[4], G2P[6], G9P[8], G12P[8]. After: G1, G2, G3, G9, G12; P[4], P[6], P[8]; G1P[8], G2P[4], G2P[6], G3P[4], G3P[8], G9P[8], G12P[8].
304	Page N, et al. (2010)	Namibia	South	1998-1999	18	cross-sectional	in and out	113	RT-PCR	Before	G1, G2, G8, G9; P[4], P[6], P[8]; G1P[4], G1P[6], G1P[8], G2P[4], G2P[6], G8P[4], G9P[6], Mix G-P.
306	Seheri LM, et al. (2010)	South Africa	South	2003-2006	48	cross-sectional	in and out	648	semi-nested, RT-PCR	Before	G1, G2, G3, G8, G9, G12; P[4], P[6], P[8]; G1P[4], G1P[6], G1P[8], G2P[4], G2P[6], G3P[4], G3P[6], G3P[8], G8P[4], G8P[6], G8P[8], G9P[4], G9P[6], G9P[8], G12P[6], Mix G-P.

Table S3. Vaccine Introduction Dates- African Countries (October 2023)

Country	Intro.Date	Vaccine	Schedule
Algeria	N/A	N/A	N/A
Angola	April 28, 2014	ROTARIX (RV1)	2+0
Benin	December 1, 2019	ROTAVAC (RV1)	3+0
Botswana	July 3, 2012	ROTARIX (RV1)	2+0
Burkina Faso	October 31, 2013	ROTASIIL (RV5)	3+0
Burundi	December 16, 2013	ROTARIX (RV1)	2+0
Cabo Verde	N/A	N/A	N/A
Cameroon	March 28, 2014	ROTARIX (RV1)	2+0
Central African Republic	N/A	ROTARIX (RV1)	2+0
Chad	N/A	N/A	N/A
Comoros	N/A	N/A	N/A
Congo	April 24, 2014	ROTARIX (RV1)	2+0
Congo, Democratic Republic of the	October 1, 2019	ROTASIIL (RV5)	3+0
Côte d'Ivoire	March 1, 2017	RotaTeq (RV5)	3+0
Equatorial Guinea	N/A	N/A	N/A
Eritrea	August 14, 2014	ROTARIX (RV1)	2+0
Eswatini	May 12, 2015	ROTARIX (RV1)	2+0
Ethiopia	November 7, 2013	ROTARIX (RV1)	2+0
Gabon	N/A	N/A	N/A
Gambia	August 14, 2013	ROTARIX (RV1)	2+0
Ghana	April 26, 2012	ROTAVAC (RV1)	2+0
Guinea	N/A	N/A	N/A
Guinea-Bissau	November 24, 2015	ROTARIX (RV1)	2+0
Kenya	July 1, 2014	ROTARIX (RV1)	2+0
Lesotho	December 18, 2017	ROTARIX (RV1)	2+0

Liberia	April 25, 2016	ROTARIX (RV1)	2+0
Marocco	October 20, 2010	ROTARIX (RV1)	2+1
Madagascar	May 5, 2014	ROTARIX (RV1)	2+0
Malawi	October 29, 2012	ROTARIX (RV1)	2+0
Mali	January 14, 2014	RotaTeq (RV5)	3+0
Mauritania	December 6, 2014	ROTARIX (RV1)	2+0
Mauritius	May 1, 2015	ROTARIX (RV1)	2+0
Mozambique	September 4, 2015	ROTARIX (RV1)	2+0
Namibia	November 11, 2014	ROTARIX (RV1)	2+0
Niger	August 5, 2014	ROTARIX (RV1)	2+0
Nigeria	August 22, 2022	ROTAVAC (RV1)	N/A
Rwanda	May 25, 2012	ROTARIX (RV1)	2+0
Sao Tome and Principe	September 22, 2016	RotaTeq (RV5)	3+0
Senegal	November 28, 2014	ROTARIX (RV1)	2+0
Seychelles	September 1, 2017	ROTARIX (RV1)	2+0
Sierra Leone	March 28, 2014	ROTARIX (RV1)	2+0
South Africa	August 1, 2009	ROTARIX (RV1)	2+0
South Sudan	N/A	N/A	N/A
Tanzania, United Republic of	December 6, 2012	ROTARIX (RV1)	2+0
Togo	June 19, 2014	ROTARIX (RV1)	2+0
Uganda	June 26, 2018	ROTARIX (RV1)	2+0
Zambia	November 26, 2013	ROTARIX (RV1)	2+0
Zimbabwe	May 1, 2014	ROTARIX (RV1)	2+0

Figure S1. Critical Appraisal of the Included Studies *

PMID	Author (DP)	DP	1. Were the criteria for inclusion in the sample clearly defined?				2. Were the study subjects and the setting described in detail?				3. Was the exposure measured in a valid and reliable way? (RV-A)				4. Were objective, standard criteria used for measurement of the condition? (Diarreia)				5. Were confounding factors identified (Limitations)?				6. strategies to deal with (limitations/ Next Steps) confounding factors stated?				7. Were the outcomes measured in a valid and reliable way? (Genotyping Methods /Genotypes Identified)				8. Was appropriate statistical analysis used? (data Organization/presentation)				
			Yes	No	Unclear	NA*	Yes	No	Unclear	NA*	Yes	No	Unclear	NA*	Yes	No	Unclear	NA*	Yes	No	Unclear	NA*	Yes	No	Unclear	NA*	Yes	No	Unclear	NA*	Yes	No	Unclear	NA*	Overall Appraisal
10022804	Cunliffe NA, et al. (1999)	1999	1				1				1				1				1				1				1				1				8
10512431	Naficy AB, et al. (1999)	1999	1				1				1				1				1				1				1				1				8
11130890	Armah GE, et al. (2001)	2001	1				1				1				1				1				1				1				1				8
11326029	Asmah RH, et al. (2001)	2001	1				1				1				1					0				0			1				1				6
11230392	Cunliffe NA, et al. (2001)	2001	1				1				1				1					0				0			1				1				6
12022161	Audu R, et al. (2002)	2002	1				1				1				1					0				0			1				1				6
12950670	Binka FN, et al. (2003)	2003	1				1				1				1						0,5			0			1				1				6,5
16087119	Nielsen NM, et al. (2005)	2005	1				1				1				1				1				1				1				1				8
17516524	Chouikha A, et al. (2007)	2007	1				1				1				1				1				1				1				1				8
19817617	Benhafid M, et al. (2009)	2009	1				1				1				1						0,5			0			1				1				6,5
20684720	Armah GE, et al. (2010)	2010	1				1				1				1					0				0			1				1				6
20684698	Cunliffe NA, et al. (2010)	2010	1				1				1				1				1				1				1				1				8
20684704	Esona MD, et al. (2010)	2010	1				1				1				1					0				0			1				1				6
20684702	Kabue JP, Peenze I, de Beer [illegible]	2010	1				1				1				1				1				1				1				1				8
20684714	Matson DO, Abdel-Messih IA	2010	1				1				1				1					0				0			1				1				6
20684701	Nyangao J, Page N, Esona M,	2010	1				1				1				1				1				1				1				1				8
20684697	Page N, Pager C, Steele AD	2010	1				1				1				1				1				1				1				1				8
20684694	Seheri LM, Page N, Dewar JB	2010	1				1				1				1				1				1				1				1				8
20684707	Steele AD, Page N, de Beer M	2010	1				1				1				1					0				0			1				1				6
20684712	Trabelsi A, Fodha I, Chouikha	2010	1				1				1				1					0				0			1				1				6
21678452	Bonkoungou IJ, Damanka S, S	2011	1				1				1				1				1				1				1				1				8
19896286	Chouikha A, Fredj MB, Fodha	2011	1				1				1				1					0				0			1				1				6
21264877	Jere KC, Sawyerr T, Seheri LM	2011	1				1				1				1				1				1				1				1				8

22964045	Nordgren J, Bonkoungou IJ, N	2012	1				1				1				1				1				1				1				1				8
22469076	Nordgren J, Nitiema LW, Sha	2012	1				1				1				1					0			1				1				1				7
23141818	Soltani M, Bouanene I, Trabe	2012	1				1				1				1					0				0			1				1				6
23074038	Benhafid M, Elomari N, Elqaz	2013	1				1				1				1				1				1				1				1				8
24034588	Enweronu-Laryea CC, Sagoe	2013	1				1				1				1				1				1				1				1				8
23765785	Ndze VN, Papp H, Achidi EA,	2013	1				1				1				1				1				1				1				1				8
23485495	Turner A, Ngwira B, Witte D,	2013	1				1				1				1					0				0			1				1				6
24343610	Abebe A, Teka T, Kassa T, Sel	2014	1				1				1				1				1				1				1				1				8
24343617	Ahmed SF, Mansour AM, Kle	2014	1				1				1				1				1					0			1				1				7
25193563	Banga-Mingo V, Waku-Kouor	2014	1				1				1				1				1				1				1				1				8
25220619	Boula A, Waku-Kouomou D, I	2014	1				1				1				1				1				1				1				1				8
24894194	El Qazoui M, Oumzil H, Baass	2014	1				1				1				1					0				0			1						0,5		5,5
24859323	Hokororo A, Kidenya BR, Sen	2014	1				1				1				1					0				0			1				1				6
24343620	Kiulia NM, Nyaga MM, Seher	2014	1				1				1				1				1				1				1				1				8
24343613	Mukaratirwa A, Berejena C, N	2014	1				1				1				1				1				1				1				1				8
24343612	Odiit A, Mulindwa A, Naluma	2014	1				1				1				1				1				1				1				1				8
24655441	Page AL, Jusot V, Mamaty AA	2014	1				1				1				1				1				1				1				1				8
24637513	Pukuta ES, Esona MD, Nkong	2014	1				1				1				1				1				1				1				1				8
25772575	Ianiro G, Delogu R, Baba M, C	2015	1				1				1				1				1				1				1				1				8
25283609	Istrate C, Sharma S, Nordgren	2015	1				1				1				1					0					0,5		1						0,5		6
25733783	Raini SK, Nyangao J, Kombich	2015	1				1				1				1				1				1				1				1				8
	Ayolabi CI	2016	1				1				1				1					0				0			1				1				6
26724820	Bwogi J, Malamba S, Kigozi B	2016	1				1				1				1				1					0			1				1				7
26370427	Damanka S, Adiku TK, Armah	2016	1				1				1				1				1						0,5		1				1				7,5
	Elnady HG, Abdelsamie OM,	2016	1				1				1				1					0				0			1				1				6
26946356	Esteves A, Nordgren J, Perei	2016	1				1				1				1					0			1				1				1				7
26378607	Mayindou G, Ngokana B, Sidi	2016	1				1				1				1					0			1				1				1				7
27375269	Moussa A, Ben Hadj Fredj M,	2016	1				1				1				1					0			1				1				1				7
27098941	Uzoma EB, Chukwubuikem C	2016	1				1				1				1					0				0			1				1				6
28422995	Gasparinho C, Piedade J, Mir	2017	1				1				1				1				1				1				1				1				8
28735327	Saudy N, Elshabrawy WO, Me	2017	1				1				1				1				1				1				1				1				8
27648929	Wandera EA, Mohammad S, I	2017	1				1				1				1					0			1				1				1				7

	Allayeh AK, El Baz RM, Saeed	2018	1	1	1	1	1		1			1	1		8
29699581	Boni-Cisse C, Meite S, Mlan A	2018	1	1	1	1		0	1			1	1		7
29718582	Bonkoungou IJO, Ouédraogo	2018	1	1	1	1	1		1			1	1		8
30138709	Gelaw A, Pietsch C, Liebert U	2018	1	1	1	1	1		1			1	1		8
28906005	Japhet MO, Famurewa O, Itu	2018	1	1	1	1	1		1			1	1		8
29052059	João ED, Strydom A, O'Neill H	2018	1	1	1	1	1		1			1	1		8
29371014	Lartey BL, Damanka S, Dennis	2018	1	1	1	1	1		1			1	1		8
29304830	Moure UAE, Banga-Mingo V,	2018	1	1	1	1	1		1			1	1		8
29628149	Mukaratirwa A, Berejena C, N	2018	1	1	1	1	1		1			1	1		8
29432666	Wandera EA, Mohammad S,	2018	1	1	1	1	1		1			1	1		8
WOS:0005	Amadu DO, Abdullahi IN, Em	2019	1	1	1	1	1			0		1	1		7
31237533	Bennour H, Fodha I, Bouazizi	2019	1	1	1	1		0		0		1	1		6
31286526	Letsa V, Damanka S, Dennis F	2019	1	1	1	1		0			0,5	1		0,5	6
31530468	Mokomane M, Esona MD, Bo	2019	1	1	1	1	1		1			1	1		8
33076976	Agbla JM, Esona MD, Agbank	2020	1	1	1	1	1		1			1		0,5	7,5
32824938	João ED, Munlela B, Chissaqu	2020	1	1	1	1	1		1			1	1		8
32998792	Rönnelid Y, Bonkoungou IJO,	2020	1	1	1	1	1				0,5	1	1		7,5
34358275	Chissaque A, Bauhofer AFL, C	2021	1	1	1	1	1		1			1	1		8
33539399	Simwaka J, Seheri M, Mulund	2021	1	1	1	1		0			0,5	1		0,5	6
35062336	Manjate F, João ED, Chirinda	2022	1	1	1	1	1		1			1	1		8

* some cut-off score, such top scores high quality (7-8), medium (6-5) and low quality (<5)

Yes= 1 No= 0 Unclear =0,5
